# Opposing roles of endothelial and leukocyte-expressed IL-7Rα in the regulation of psoriasis-like skin inflammation

**DOI:** 10.1038/s41598-019-48046-y

**Published:** 2019-08-12

**Authors:** Martina Vranova, Mona C. Friess, Neda Haghayegh Jahromi, Victor Collado-Diaz, Angela Vallone, Olivia Hagedorn, Maria Jadhav, Ann-Helen Willrodt, Anna Polomska, Jean-Christophe Leroux, Steven T. Proulx, Cornelia Halin

**Affiliations:** Institute of Pharmaceutical Sciences, ETH Zurich, Zurich, Switzerland

**Keywords:** Experimental models of disease, Lymphatic vessels

## Abstract

The interleukin 7 receptor alpha chain (IL-7Rα) is predominately expressed by lymphocytes, and activation by its ligand IL-7 supports the development and maintenance of T cells and boosts T-cell mediated immunity. We recently reported that lymphatic endothelial cells (LECs) in dermal lymphatics also express IL-7 and its receptor chains (IL-7Rα and CD132) and that IL-7 supports lymphatic drainage. This suggested that activation of IL-7Rα signaling in lymphatics could exert inflammation-resolving activity, by promoting the clearance of excess tissue fluid. Here we investigated how the potentially opposing effects of IL-7Rα signaling in immune cells and in the lymphatic vasculature would affect the development and progression of psoriasis-like skin inflammation. We found that during acute and chronic skin inflammation mice with an endothelial-specific deletion of IL-7Rα (IL-7Rα^ΔEC^ mice) developed more edema compared to control mice, as a consequence of impaired lymphatic drainage. However, systemic treatment of wild-type mice with IL-7 exacerbated edema and immune cell infiltration in spite of increasing lymphatic drainage, whereas treatment with IL-7Rα blocking antibody ameliorated inflammatory symptoms. These data identify IL-7Rα signaling as a new pathway in psoriasis-like skin inflammation and show that its pro-inflammatory effects on the immune compartment override its anti-inflammatory, drainage-enhancing effects on the endothelium.

## Introduction

Afferent lymphatic vessels are present in virtually all tissues of the body and play important roles in the regulation of tissue fluid homeostasis, inflammation and immunity. During an inflammatory response, blood vessels become permeable leading to the extravasation of blood-derived fluids, macromolecules and leukocytes into the tissue. Lymphatic vessels serve to take up excess fluid as well as immune mediators and leukocytes to facilitate their removal from the inflamed tissue and transport to draining lymph nodes (LNs) or beyond. In doing so, lymphatic vessels exert important anti-inflammatory functions, as also apparent in lymphedema, where lymphatic dysfunction induces chronic inflammatory changes in the affected skin^1^. Animal studies have recently suggested that stimulation of lymphangiogenesis and lymphatic drainage, to remove excess fluids (i.e. edema) and inflammatory mediators from the tissue, may offer a novel approach for the treatment of chronic inflammatory diseases: Specifically, activation of vascular endothelial growth factor (VEGF) receptor 3 (VEGFR-3) by VEGF-C was shown to accelerate the resolution of rheumatoid arthritis^2^ and inflammatory bowel disease^3^ and to ameliorate acute and chronic skin inflammation^4–6^ in various mouse models^7^.

We have recently reported that similarly to VEGF-C, interleukin-7 (IL-7) supports lymphatic drainage function *in vivo*^8^. IL-7 is a pleiotropic cytokine, which is primarily produced by stromal cells in primary and secondary lymphoid organs, keratinocytes and epithelial cells^9^. It is important for T and B cell development, lymph node (LN) organogenesis and the survival of naive and memory T cells^9,10^. Moreover, IL-7 was found to promote effector functions of T helper (Th)1 and Th17 cells and of cytotoxic T cells^11–14^. We recently reported that dermal lymphatic endothelial cells (LECs) express both IL-7 as well as its two receptor chains, i.e. the IL-7 receptor alpha chain (IL-7Rα) and the common cytokine receptor gamma chain (CD132). Treatment with IL-7 supported lymphangiogenic processes *in vitro*, and mice with a global deletion of IL-7Rα (IL-7Rα^−/−^) or overexpression of IL-7 in all cell types (IL-7tg mice) displayed alterations in their lymphatic network in the skin and the diaphragm (i.e. the two organs analysed)^8^. We observed that drainage through dermal lymphatic vessels was reduced in IL-7Rα^−/−^ mice, while it was enhanced in IL-7tg mice, or upon treatment with recombinant IL-7/anti-IL-7 complexes^8^. Our study further revealed that the drainage-enhancing activity of IL-7 was dependent on IL-7Rα expression in stromal cells, indicating a direct autocrine action of IL-7 on LECs^8^.

Enhancing lymphatic drainage by activation of the IL-7Rα signaling pathway could therefore – in analogy to VEGF-C^4^ – represent a novel approach for reducing tissue inflammation. However, in contrast to VEGF-C, IL-7 has also been linked to the pathogenesis of a number of autoimmune diseases. Levels of IL-7 or of a soluble form of the IL-7Rα reportedly are upregulated in various autoimmune diseases (either in serum or at the site of inflammation)^13,15^ and polymorphisms in the IL-7Rα gene have been associated with the development of multiple sclerosis or type I diabetes^13,15–17^. Conversely, treatment with an IL-7-blocking antibody was shown to confer a therapeutic benefit in a mouse model of collagen-induced arthritis (CIA)^18^, while treatment with IL-7Rα blocking antibodies reduced inflammation in mouse models of type 1 diabetes^19,20^, CIA^21^, or multiple sclerosis^22^. Based on these findings, an IL-7Rα blocking antibody has recently entered clinical development for the treatment of autoimmune conditions^23^.

Psoriasis is an autoimmune disorder for which the contribution of the IL-7/IL-7Rα signalling pathway has not been investigated to date. This disease is triggered by skin-infiltrating autoreactive Th17 cells, which produce high levels of inflammatory mediators^24^, leading to (lymph)angiogenesis, aberrant keratinocyte differentiation/proliferation and the formation of characteristic erythematous plaques. In the skin, IL-7 is constitutively produced by epidermal keratinocytes^25^ as well as by dermal lymphatics^8^. Interestingly, several studies have previously reported that IL-7 levels were increased in psoriatic skin and/or in the plasma of psoriasis patients^26–28^. Since edema formation is one of the cardinal signs of inflammation and our previous findings had demonstrated that the IL-7/IL-7Rα axis supports lymphatic drainage, we here set out to further investigate the overall role of IL-7Rα signaling in psoriasis-like skin inflammation. Specifically, we investigated in three different mouse models how the potentially opposing effects of IL-7Rα signaling on immune cells (i.e. immune-stimulating) and on the lymphatic vasculature (i.e. drainage-enhancing and inflammation-resolving) would affect the development, and progression of psoriasis-like skin inflammation. Our results identify the IL-7Rα signaling axis as a new disease-promoting pathway in psoriasis and show that in the context of chronic skin inflammation the pro-inflammatory effects of IL-7Rα signaling in the immune compartment override its inflammation-resolving and lymphatic drainage-enhancing effects on the endothelium.

## Results

### Endothelial-specific deletion of IL-7Rα enhances edema formation and decreases lymphatic drainage in TPA-induced acute skin inflammation

**Figure 1:**
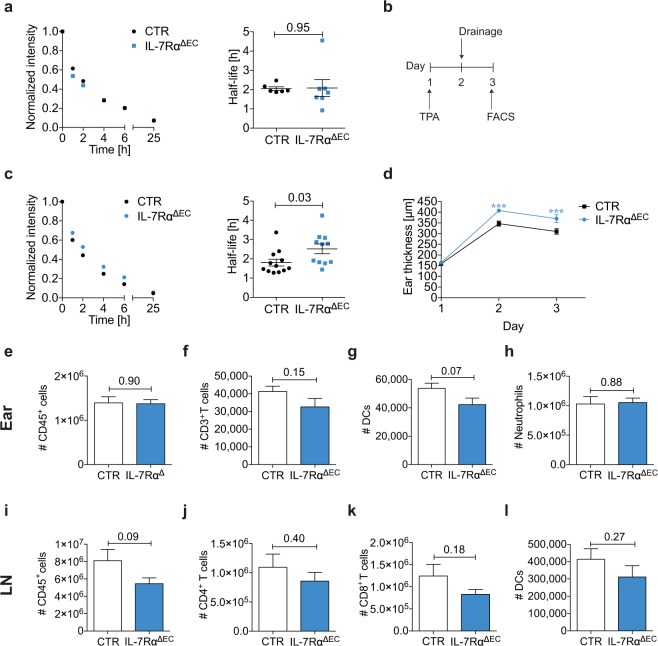
Endothelial-specific deletion of IL-7Rα decreases lymphatic drainage and increases edema formation in TPA-induced inflammation. (**a**) IL-7Rα^ΔEC^ and control (CTR) mice were injected with a P20D800 conjugate i.d. in the uninflamed ear skin. Clearance of the tracer was monitored over 24 hours using an IVIS imaging system. Average clearance plots of P20D800 (left) and calculated half-lives (right) in CTR and IL-7Rα^−/−^ mice (data from 1 experiment with 6 mice per group). (**b**) TPA (black arrow) was applied to the ears of IL-7Rα^ΔEC^ and CTR mice on day 1. (**c**) Average clearance plots of P20D800 (left) and calculated half-lives (right) on day 2 after TPA, assessed as in (A). (**d**) Ear thickness measurements between days 1–3. *** p < 0.001. FACS-based quantification of the number of (**e**,**i**) CD45^+^ cells, (**f**) CD3^+^ T cells, (**g**–**l**) DCs, (**h**) neutrophils, (**j**) CD4^+^ T cells, (**k**) CD8^+^ T cells in the ear skin (**e**–**h**) and dLNs (**i**–**l**) on day 3. Per animal, both ears and both ear-dLNs were pooled for the FACS analysis. Pooled data (mean ± SEM) from 2 similar experiments with a total of 11–12 mice per group are shown for (**b**–**l**).

In our previous work, we had quantified lymphatic drainage function by measuring the levels of Evans blue that were retained in the murine ear skin at a defined time point after intradermal (i.d.) injection^8^. In this study, we assessed lymphatic drainage from the ear skin with a more dynamic method, namely by measuring the disappearance of an i.d. injected PEGylated infrared dye (P20D800) over 24 hours^29^. In line with our previous findings^8^, we observed decreased lymphatic drainage and an increased half-life in IL-7Rα^−/−^ compared to wild type (WT) mice (Suppl. Fig. 1). Our previous study had indicated that the effect of IL-7Rα signaling on lymphatic drainage was dependent on IL-7Rα expression in stromal cells^8^. To specifically test whether this effect involved the expression of IL-7Rα in endothelial cells, we created a mouse with an endothelial-specific deletion of the IL-7Rα (IL-7Rα^ΔEC^). To this end, we crossed mice that express the cre recombinase under the vascular endothelial (VE)-cadherin promotor (VE-cadherin-cre mice^30^) with IL-7Rα^fl/fl^ mice^31^. qPCR analysis performed on DNA extracted from isolated LN LECs of IL-7Rα^ΔEC^ mice (VE-cadherin^cre/cre^ x IL-7Rα^fl/fl^) and control mice (VE-cadherin^wt/wt^ x IL-7Rα^fl/fl^, derived from the same line) suggested deletion rates of approx. 97% (Suppl. Fig. 2) Similarly, fluorescence-activated cell sorting (FACS) analysis performed on LN single-cell suspensions indicated the absence of IL-7Rα (CD127) protein from LECs and blood endothelial cells (BECs) while it remained expressed in e.g. T cells (Suppl. Fig. 3). This analysis also confirmed our previous findings^8^ that, in comparison to IL-7Rα levels on T cells, expression in LECs and BECs is very low (Suppl. Fig. 3). No significant differences were observed in steady-state leukocyte populations analyzed by FACS performed on singe-cell suspensions in the ear skin or LNs of IL-7Rα^ΔEC^ mice (Suppl. Figs 4 and 5). Analysis of lymphatic drainage did not reveal any differences in drainage under steady-state conditions (Fig. 1a), but drainage was significantly decreased in IL-7Rα^ΔEC^ mice as compared to controls during acute inflammation induced by topical application of 12-O-tetradecanoyl phorbol-13-acetate (TPA) (Fig. 1b,c, Suppl. Fig. 6a). Notably, while a minute FACS signal for expression was observed in dermal LECs and BECs of TPA-inflamed control mice, this was consistently reduced in IL-7Rα^ΔEC^ mice (Suppl. Fig. 6b–e). In line with reduced drainage, we also observed that TPA-induced inflammation led to a more profound ear swelling response in IL-7Rα^ΔEC^ as compared to control mice (Fig. 1d). However, in spite of the edematous and seemingly more inflamed appearance, FACS analysis performed on singe-cell suspensions did not reveal any increase in leukocyte numbers in ear skin of IL-7Rα^ΔEC^ compared to control mice (Fig. 1e–h). A trend towards decreased overall cellularity, T cell and DC numbers was seen in ear-draining auricular LNs of IL-7Rα^ΔEC^ mice (Fig. 1i–l). Overall, these findings showed that during acute skin inflammation, loss of endothelial-cell expressed IL-7Rα reduced lymphatic drainage and exacerbated edema formation, i.e. one of the cardinal signs of inflammation.

### mIL-7-Fc treatment exacerbates oxazolone-induced skin inflammation in K14-VEGF-A mice in spite of increased lymphatic drainage

**Figure 2:**
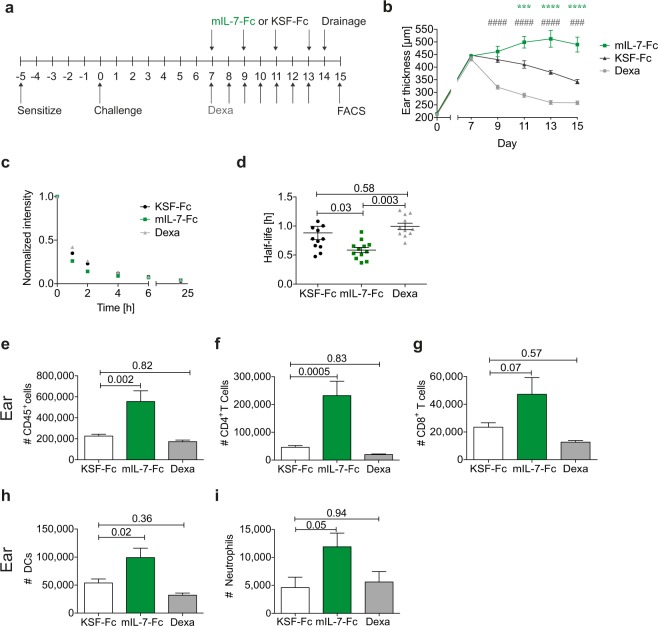
mIL-7-Fc treatment exacerbates oxazolone-induced inflammation in K14-VEGF-A mice in spite of increasing lymphatic drainage. (**a**) Hemizygous K14-VEGF-A mice were sensitized with oxazolone on the belly and paws. Five days later the ears were challenged with oxazolone. Mice were randomized into treatment groups of comparable ear thickness on day 7 and treated i.p. with mIL-7-Fc, KSF-Fc every second day, or with dexamethasone (Dexa) every day for one week. The ear thickness was measured on each treatment day. Lymphatic drainage was measured on day 14 after challenge, and the mice were sacrificed on day 15. Immune cell infiltration in the ear skin was analyzed by FACS. (**b**) Ear thickness measurements over the course of the treatment. P values indicated by an asterisk show comparisons between mIL7-Fc and KSF-Fc, and p values indicated by a pound sign show comparisons between Dexa and KSF-Fc. ^###/^***p < 0.001; ^####/^****p < 0.0001. (**c**,**d**) Lymphatic drainage analysis: (**c**) Average clearance plots of P20D800 measured by IVIS and (**d**) the calculated half-lives. FACS-based quantification of the number of (**e**) CD45^+^ cells, (**f**) CD4^+^ T cells, (**g**) CD8^+^ T cells, (**h**) DCs, (**i**) neutrophils in the ear skin on day 15. One ear per mouse was used for the FACS analysis. Pooled data (mean ± SEM) from 2 similar experiments with a total of 11–12 mice per group are shown.

We next set out to investigate the systemic impact of IL-7Rα signaling on chronic, psoriasis-like skin inflammation. In our previous study we had pharmacologically treated wild-type (WT) mice with IL-7/anti-IL-7 complexes to enhance drainage of dermal lymphatics in steady-state^8^. As an alternative, more convenient tool for activating IL-7Rα signaling, we now generated a murine IL-7 Fc fusion protein (mIL-7-Fc) by fusing the sequence encoding mIL-7 with the hinge and Fc regions of murine immunoglobulin 1 (IgG_1_) (Suppl. Fig. 7a–c). The biological activity of mIL-7-Fc was confirmed *in vitro* and *in vivo* by testing its ability to induce T cell proliferation and survival (Suppl. Fig. 7d–g). In comparison to IL-7/anti-IL-7 complexes, approximately ten times (10x) higher molar equivalents of mIL-7-Fc were required to achieve the same degree of CD45^+^ leukocyte expansion in LNs and spleen, in line with previous reports of a similar IL-7-Fc fusion protein^32^. Therefore, this dose (mIL-7-Fc (10x)) was chosen for our *in vivo* inflammation studies (Suppl. Fig. 7e–g). Next, we investigated the effect of mIL-7-Fc treatment on the course of skin inflammation in K14-VEGF-A mice. The latter represent a well-recognized mouse model of psoriasis^4,6,33–36^. Homozygous K14-VEGF-A mice spontaneously develop inflammatory skin lesions at few weeks of age^35^. By contrast, in hemizygous mice, psoriasis-like chronic skin inflammation that persists over several weeks can be initiated by the induction of a contact hypersensitivity (CHS) response to oxazolone. At the site of challenge, i.e. the mouse ear, the inflamed skin is characterized by inflammatory cell infiltration, epidermal hyperproliferation, vascular expansion, and edema formation (Suppl. Fig. 8a)^4,34,35^. Accordingly, hemizyguous K14-VEGF-A mice were sensitized and challenged with oxazolone. Treatment with mIL-7-Fc was started seven days after challenge and continued for eight days (Fig. 2a, Suppl. Fig. 8b–e). Control groups were either treated with KSF-Fc, i.e. an Fc-fusion of a single-chain variable fragment (scFv) directed against hen egg lysozyme^37^, or dexamethasone (i.e. a corticosteroid). While dexamethasone effectively reduced the inflammation-induced ear swelling response, treatment with mIL-7-Fc increased ear thickness with respect to the KSF-Fc control group, indicative of an exacerbation of the inflammatory response (Fig. 2b). However, at the same time, lymphatic drainage improved significantly in mIL-7-Fc-treated mice compared to the KSF-Fc- and dexamethasone-treated groups (Fig. 2c,d). The increased ear thickness in the mIL-7-Fc treated mice compared to the KSF-Fc treated group was accompanied by an increase in the infiltration of CD45^+^ cells, such as CD4^+^ and CD8^+^ T cells (Fig. 2e–g), DCs and neutrophils (Fig. 2h,i) (for the FACS gating scheme see Suppl. Fig. 4). In addition, the exacerbated inflammation in the ear skin seen upon IL-7-Fc treatment led to a further expansion of the lymphatic and blood vessel area compared to the control groups (Suppl. Fig. 9a–c). By contrast, analysis of keratin 6 and keratin 10 expression revealed no further exacerbation of epidermal thickening in the IL-7-Fc-treated group compared to the KSF-Fc-treated group (Suppl. Fig. 9d–f). Overall, these data indicated that mIL-7-Fc increased lymphatic drainage, but that the global stimulation of the immune response overrode this anti-inflammatory effect and resulted in exacerbated inflammation with a higher level of edema and immune cell infiltration.

### Anti-IL-7Rα treatment ameliorates oxazolone-induced skin inflammation in K14-VEGF-A mice

**Figure 3:**
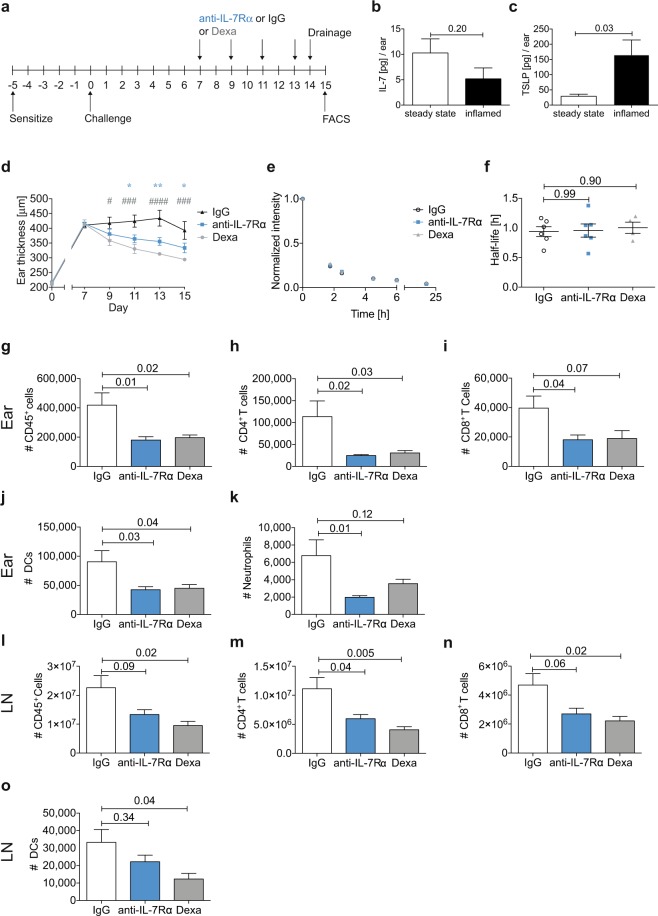
Anti-IL-7Rα treatment ameliorates oxazolone-induced inflammation in K14-VEGF-A mice. (**a**) Hemizygous K14-VEGF-A mice were sensitized with oxazolone on the belly and paws. Five days later their ears were challenged with oxazolone. Mice were randomized into treatment groups on day 7 and treated i.p. with anti-IL-7Rα antibody, corresponding IgG control, or with dexamethasone (Dexa) on days 7, 9, 11, and 13. The ear thickness was measured on each treatment day. Lymphatic drainage was measured on day 14 after challenge, and the mice were sacrificed on day 15. Immune cell infiltration in the ear skin and dLNs was analyzed by FACS. (**b**) IL-7 and (**c**) TSLP protein levels were measured from ear skin lysates on day 8 (n = 4 per group). (**d**) Ear thickness measurements over the course of the treatment. P values indicated by an asterisk show comparisons between anti-IL-7Rα and IgG. P values indicated by a pound sign show comparisons between Dexa and IgG. ^#/^*p < 0.05; **p < 0.01 ^**###**^p < 0.001; ^**####**^p < 0.0001. (**e**,**f**) Lymphatic drainage analysis: (**e**) Average clearance plots of P20D800 from the ear measured by IVIS and (**f**) the calculated half-lives. FACS-based quantification of the number of (**g**,**l**) CD45^+^ cells, (**h**,**m**) CD4^+^ T cells, (**i**,**n**) CD8^+^ T cells, (**j**,**o**) DCs, (**k**) neutrophils measured in the ear skin (**g**–**k**) and in dLNs (**l**–**o**) on day 15. One ear per mouse and both dLNs were used for the FACS analysis. Data (mean ± SEM) from 1 out of 3 similar experiments (**d**,**g**–**k**) and data from 1 out of 2 experiments with each 5–7 mice per group (**e**,**f**,**l**–**q**) are shown.

Since mIL-7-Fc exacerbated edema and immune cell infiltration, we hypothesized that treatment with an anti-IL-7Rα blocking antibody might ameliorate the inflammatory symptoms in the K14-VEGF-A model. To address this, we first measured the levels of known ligands of the IL-7Rα signaling pathway in the inflamed skin (Fig. 3a–c). Besides IL-7, thymic stromal lymphopoietin (TSLP), which is produced by dermal keratinocytes, is a second cytokine that signals through the IL-7Rα (when combined with the specific TSLPR chain)^38,39^. TSLP contributes to Th2 immunity and plays an important role in human allergic diseases^38,39^, but was also found to be upregulated in psoriatic skin lesions^40^. Interestingly, we found that also the TSLPR is expressed by murine LECs *in vivo*, and that TSLP induced lymphangiogenic processes in cultured human LECs *in vitro* (Suppl. Fig. 10). While there was no significant change in IL-7 levels (Fig. 3b), TSLP protein levels were profoundly increased in day 8-inflamed skin (Fig. 3c). CD4^+^ effector/memory T cells in the inflamed ear skin expressed high levels of IL-7Rα, whereas its expression in the vasculature was very low (Suppl. Fig. 8b–e). Next, we investigated how IL-7Rα blockade would affect the progression of inflammation. Treatment with anti-IL-7Rα blocking antibody ameliorated edema in comparison to the IgG-treated group (Fig. 3d), while lymphatic drainage remained unchanged (Fig. 3e,f). In comparison to the IgG-treated group, infiltration of leukocytes (Fig. 3g–k) was similarly reduced in both the dexamethasone and the anti-IL-7Rα-treated groups. As a further indication of the down-modulation of the inflammatory response, the cellularity of the dLNs was reduced in the anti-IL-7Rα-treated compared to the IgG-treated group (Fig. 3l–o, FACS gating see Suppl. Fig. 4). In addition, treatment with either anti-IL-7Rα or dexamethasone induced a normalization of the vasculature in the inflamed ear skin (Suppl. Fig. 11). In combination with our findings showing that mIL-7Fc exacerbates inflammatory symptoms in spite of increased drainage, these data suggest that the effects of IL-7Rα signaling on the immune compartment are dominant over the effects on lymphatic drainage.

### Anti-IL-7Rαtreatment ameliorates imiquimod-induced inflammation

**Figure 4:**
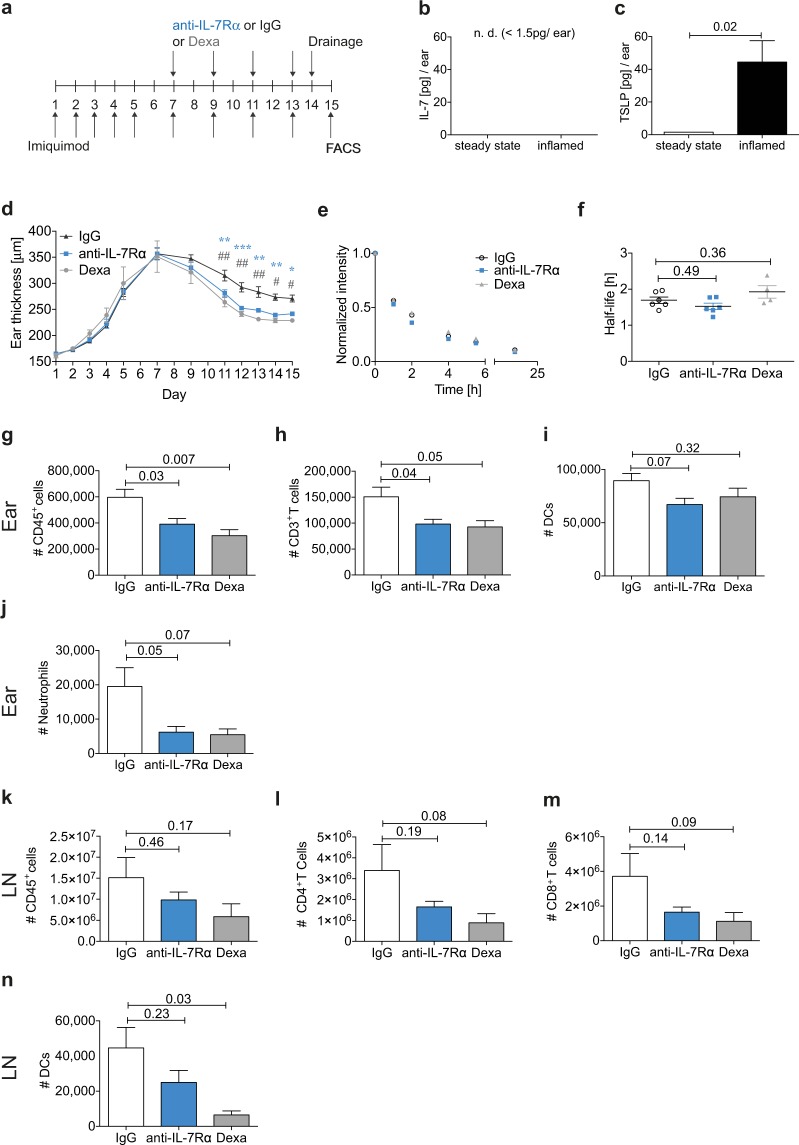
Anti-IL-7Rα treatment ameliorates imiquimod-induced inflammation in WT mice. (**a**) Imiquimod-containing cream was applied to the ears on day 1, 2, 3, 4, 5, 7, 9, 11, and 13. Mice were randomized into treatment groups on day 7 and treated i.p. with anti-IL-7Rα antibody, corresponding IgG control, or with dexamethasone (Dexa) on days 7, 9, 11, and 13. The ear thickness was measured on each treatment day. Lymphatic drainage was measured on day 14 and the mice were sacrificed on day 15. Immune cell infiltration in the ear skin and dLNs was analyzed by FACS. (**b**) IL-7 and (**c**) TSLP protein levels were measured from ear skin lysates on day 8. (**d**) Ear thickness measurements over the course of the treatment. P values indicated by an asterisk show comparisons between anti-IL-7Rα and IgG. P values indicated by a pound sign show comparisons between Dexa and IgG. ^#/^*p < 0.05; ^##/^**p < 0.01; ***p < 0.001. (**e**,**f**) Lymphatic drainage analysis: (**e**) Average clearance plots of P20D800 from the ear measured by IVIS and (**f**) the calculated half-lives. FACS-based quantification of the number of (**g**,**k**) CD45^+^ cells, (**h**) CD3^+^ T cells, (**i**,**n**) DCs, (**j**) neutrophils, (**l**) CD4^+^ T cells, (**m**) CD8^+^ T cells measured in the ear skin (**g**–**j**) and dLNs (**k**–**n**) on day 15. Both ears and dLNs were pooled for the FACS analysis. Data (mean ± SEM) from 1 experiment with 4–6 mice per group are shown.

Next, we tested whether the anti-IL-7Rα treatment would also ameliorate inflammation in another mouse model of psoriasis. Topical application of the toll-like receptor (TLR) 7/8 agonist imiquimod induces a psoriasis-like inflammation in the skin^41^. To establish inflammation, we applied imiquimod-containing cream to the ears of C57BL/6 mice every day for five days and every second day thereafter (Fig. 4a, Suppl. Fig. 12a). Also, in imiquimod-induced inflammation, CD4^+^ effector/memory T cells in the inflamed skin expressed considerable levels of IL-7Rα (Suppl. Fig. 12b–c). Conversely, the FACS signal for IL-7Rα expression detected in LECs and BECs was extremely low, yet consistently higher in control mice as compared to IL-7Rα^ΔEC^ mice (Suppl. Fig. 12d,e). IL-7 expression was undetectable in the ear skin by ELISA, but TSLP levels were increased at the peak of imiquimod-induced inflammation on day 8 (Fig. 4b,c). Also in this model treatment with anti-IL-7Rα ameliorated edema (Fig. 4d) without detectable changes in lymphatic drainage (Fig. 4e,f). By contrast, anti-IL-7Rα treatment led to decreased immune cell infiltration in the ear (Fig. 4g–j) and a slight albeit not significant reduction in cellularity of the dLN (Fig. 4k–n). These data confirm the involvement of the IL-7Rα pathway in the pathogenesis of psoriasis in another mouse model and highlight the therapeutic potential of blocking IL-7Rα signaling.

### Endothelial-specific deletion of IL-7Rα decreases lymphatic drainage and increases edema formation in imiquimod-induced inflammation

**Figure 5:**
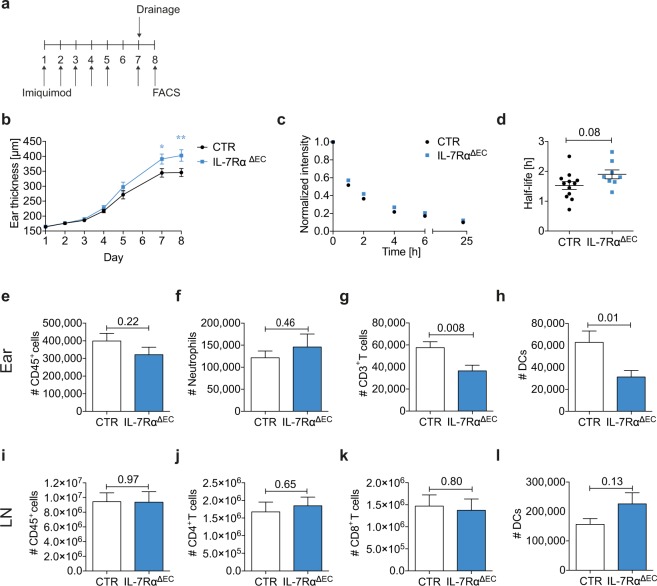
Endothelial-specific deletion of IL-7Rα decreases lymphatic drainage and increases edema formation in imiquimod-induced inflammation. (**a**) Imiquimod-containing cream was applied to the ears of IL-7Rα^ΔEC^ and control (CTR) mice on days 1–5, and 7. The ear thickness was measured on each treatment day. Lymphatic drainage was measured on day 7 after challenge and the mice were sacrificed on day 8. The immune cell infiltration in the ear skin was analyzed by FACS. (**b**) Ear thickness measurements over the course of the treatment. *p < 0.05; **p < 0.01. (**c**,**d**) Lymphatic drainage analysis: (**c**) Average clearance plots of P20D800 from the ear measured by IVIS and (**d**) the calculated half-lives. FACS-based quantification of the number of (**e**,**i**) CD45^+^ cells, (**f**) neutrophils, (**g**) CD3^+^ T cells, (**h**,**l**) DCs, (**j**) CD4^+^ T cells, (**k**) CD8^+^ T cells in (**e**–**h**) the ear skin and (**i**–**l**) dLNs on day 8. The average number of immune cells per ear is shown. Pooled data (mean ± SEM) from 3 experiments with a total of 17–18 mice per group (**b**,**e**–**l**) and pooled data from 2 experiments with 8–12 mice per group (**c**,**d**) are shown.

We showed that IL-7Rα stimulation exacerbated edema and immune cell infiltration, while IL-7Rα blockade resolved inflammatory symptoms. The effects of IL-7Rα stimulation or blockade therefore seemed to primarily be dictated by its effect on the immune response. To investigate whether loss of IL-7Rα signaling in endothelial cells would have any impact on the progression of inflammation and on lymphatic function in chronic, psoriasis-like skin inflammation, we established imiquimod-induced skin inflammation in IL-7Rα^ΔEC^ mice (Fig. 5a). IL-7Rα^ΔEC^ mice developed more severe edema in the ear compared to control mice (Fig. 5b). In line with these findings, a near-significant decrease in lymphatic drainage was observed in the ear skin of IL-7Rα^ΔEC^ mice (Fig. 5c,d). No difference in overall CD45^+^ leukocytes (Fig. 5e) or neutrophils (Fig. 5f) were seen, but CD3^+^ T cell (Fig. 5g) and DC (Fig. 5h) numbers were significantly reduced in ear skin of IL-7Rα^ΔEC^ as compared to control mice. At the same time, no significant differences in leukocyte populations in the dLNs were observed (Fig. 5i–l). This indicates that the increased edema and near-significant reduction in lymphatic drainage seen in imiquimod-induced inflammation in IL-7Rα^ΔEC^ mice was due to the effects of IL-7Rα signaling on lymphatic function.

## Discussion

In this study, we dissected the role of IL-7Rα signaling in mouse models of acute or psoriasis-like, chronic skin inflammation. We found that IL-7Rα signaling had opposing effects on endothelial cells and on leukocytes in the regulation of skin inflammation: endothelial-specific genetic deletion of the IL-7Rα resulted in increased edema formation as a consequence of impaired lymphatic drainage, while the effects on the inflammatory cell infiltrate were largely unchanged. These data illustrate the importance of lymphatic drainage for the resolution of inflammatory edema in the skin, in accordance with previous studies^4–6,42^. By contrast, when systemically enhancing or blocking IL-7Rα signaling in mice with normal IL-7Rα expression, we found that the effects on the immune compartment dominated the inflammatory response, and IL-7Rα signaling contributed to the exacerbation of psoriasis-like symptoms.

In line with our previous findings^8^, we observed that under steady-state conditions lymphatic drainage was reduced in IL-7Rα^−/−^ mice, while it was increased in mice treated with mIL-7-Fc. In the context of psoriasis-like skin inflammation, treatment with mIL-7-Fc resulted in a profound exacerbation of the inflammatory symptoms. Nevertheless, we observed an increase in lymphatic drainage upon mIL-7-Fc treatment compared to KSF-Fc and dexamethasone treatment in this model. Given that there was no correlation between lymphatic drainage and the increase in ear thickness in KSF-Fc- and dexamethasone-treated mice (which do have a significant difference ear thickness) the enhanced drainage rates observed in the mIL-7-Fc treated group cannot simply be explained by the exacerbated edema. In fact, in comparison to steady-state conditions, lymphatic drainage was reportedly decreased in oxazolone-induced inflammation in K14-VEGF-A mice^4,43^. This strongly suggests that the increase in drainage seen upon treatment with mIL-7-Fc was caused by IL-7’s drainage promoting activity. Interestingly, treatment with anti-IL-7Rα did not result in a decrease in lymphatic drainage in the two inflammation models tested (K14-VEGF-A and imiquimod model). This might be explained by the much higher IL-7Rα expression on leukocytes compared to endothelial cells, which favors antibody binding and blocking activity on leukocytes. Blocking the low IL-7Rα levels on endothelial cells and particular on LECs, which constitutively produce the IL-7Rα ligand IL-7^8^, is likely much more challenging. This failure to inhibit autocrine IL-7/IL-7Rα signaling in LECs might therefore explain why drainage was not reduced in anti-IL-7Rα-treated mice. Of interest, autocrine VEGF-A signaling in endothelial cells is known to be indispensable for endothelial survival, but to occur mainly through intracellular autocrine receptor binding and signaling^44^. Similar to our findings with anti-IL-7Rα treatment, this survival promoting function of VEGF-A was apparent in endothelial cell-specific VEGF-A knockouts but could not be observed upon treatment with anti-VEGF-A antibody^44^.

In our previous study we had observed that the drainage-promoting activity of IL-7Rα signaling depended on IL-7Rα expression in stromal cells^8^. Here, we show that genetic deletion of IL-7Rα in endothelial cells suffices to modulate lymphatic drainage. Even though no drainage defect was observed in IL-7Rα^ΔEC^ mice under steady state, drainage was decreased upon TPA and imiquimod-induced inflammation. In both TPA-induced acute and imiquimod-induced chronic inflammation, reduced drainage was accompanied by a significant increase in ear thickness (i.e. tissue edema) in IL-7Rα^ΔEC^ mice. By contrast, upon systemic treatment with anti-IL-7Rα, the ear swelling response was profoundly reduced whereas drainage was not altered. The differential effects of anti-IL-7Rα and endothelial IL-7Rα deletion on the ear swelling response can likely be explained as follows: upon systemic blockade of IL-7Rα, levels of infiltrating leukocytes were strikingly reduced both in the K14-VEGF-A and in the imiquimod-induced skin inflammation model. It is fair to assume, that in presence of fewer cellular infiltrates also less inflammatory mediators were produced, resulting in less blood vascular leakage and inflammation-induced edema. Considering that lymphatic drainage was not reduced under these conditions (likely because antibody treatment failed to block autocrine IL-7/IL-7Rα signaling in LECs), the decrease in ear thickness can mainly be attributed to treatment-induced reduction in leukocyte infiltration and activation. Conversely, in the genetic model, in which IL-7Rα was lacking in endothelial cells but leukocyte infiltration was in large parts unaffected, the reduced lymphatic drainage accounted for the increased ear thickness measured in the IL-7Rα^ΔEC^ mice.

While mIL-7-Fc exacerbated psoriasis-like inflammation, anti-IL-7Rα treatment improved edema, reduced immune cell infiltration, and decreased the cellularity of the dLN. The anti-inflammatory effects of the anti-IL-7Rα treatment are likely mediated by the blockade of IL-7Rα signaling in immune cells. It is well known that IL-7 expands the naive and memory T cell pool^9^, but in accordance with our own findings, recent studies have also shown IL-7Rα expression in effector T cells^45,46^. Moreover, there are several studies showing that IL-7 supports the expansion and function of Th1 and Th17 responses^11–14^, which are considered the main drivers of human psoriasis^24^, and also relevant in the K14-VEGF-A^33,47^ and imiquimod-induced^41^ skin inflammation models used in this study. In addition to impacting homeostasis and function of conventional αβ T cells, IL-7 is also necessary for the survival of γδ T cells^48^, which were shown to be important IL-17 producers and drivers of inflammation in the K14-VEGF-A and imiquimod-induced inflammation models^47,49^. Thus, similarly to its suggested activity in other mouse models of autoimmunity^13,19–22^, anti-IL-7Rα treatment likely reduced the Th1 and Th17 response in the psoriasis-like skin inflammation investigated here.

At present, we do not know whether the observed anti-inflammatory effects of anti-IL-7Rα treatment were entirely due to blocking IL-7 or also due to blocking TSLP. In contrast to the reported upregulation of IL-7 in human psoriasis^26,28^, we did not observe an upregulation of IL-7 in the inflamed murine ear skin. However, this does not exclude that local levels of IL-7 produced by LECs or keratinocytes in the skin were sufficient to support IL-7-mediated responses of effector T cells in the inflamed tissue. Furthermore, anti-IL-7Rα treatment likely does not only act locally, at the site of inflammation, but rather also reduces IL-7Rα signalling in secondary lymphoid organs, where high amounts of IL-7 are constantly produced by stromal cells^9^.

In agreement with a study reporting its expression in human psoriatic skin lesions^40^, TSLP was markedly upregulated in inflamed murine skin, suggesting that inflammation-induced TSLP could also modulate lymphatic drainage and the immune response. In further support of this notion, our data showed that TSLP induced lymphangiogenic processes in LECs *in vitro*. However, in contrast to IL-7, TSLP does not appear to be expressed in LECs *in vivo*^50^. Given that local concentrations of IL-7 in the vicinity of/within LECs are expected to be very high and that at the same time IL-7Rα expression on LECs is very low, it is unlikely that inflammation-induced TSLP can effectively compete with the autocrine IL-7/IL-7Rα signalling in LECs. On the other hand, TSLP could also modulate the inflammatory response, by affecting T cell polarization and effector function. TSLP is strongly linked with the induction and maintenance of Th2 responses^38,39^. However, considering that skin inflammation induced by imiquimod^41^ and in K14-VEGF-A mice^33,47^ carries a Th17 signature, it seems rather unlikely that TSLP is the main driver of the IL-7Rα-mediated inflammatory responses in our models. Furthermore, Th17 cells reportedly express much lower levels of TSLPR than Th2 cells, which are considered the primary TSLP target cells^51^. Besides its expression on T cells, also dendritic cells (DCs) express the TSLPR, and TSLP is well known for its ability to polarize DCs to induce T cell activation and differentiation into Th2 cells^38,39^. Interestingly, a recent study also reported a role for TSLP in inducing the production of the Th17-promoting cytokine IL-23 in human DCs^40^. Thus, although the current literature would suggest a more prevalent role for IL-7 thanks to its Th1- and Th17- promoting activity, a potential contribution of TSLP to psoriasis-like skin cannot be ruled out at present.

Overall, our data describe a new role for IL-7Rα signaling in the progression of psoriasis-like skin inflammation. We show for the first time that blocking IL-7Rα signaling improves psoriasis-like inflammation and might be a valuable therapeutic target. This is of particular relevance, considering that a first antibody targeting IL-7Rα is currently in clinical development^23^.

## Methods

### Mice

WT C57BL/6 mice were purchased from Janvier (Janvier, Saint-Isle, France). IL-7Rα^−/−^^8,52^ and hemizygous K14-VEGF-A transgenic mice (referred to as K14-VEGF-A)^34,35^ have been previously described. Mice with an endothelial-specific knockout of the IL-7Rα, driven by the VE-cadherin promotor (referred to as IL-7Rα^ΔEC^ mice), were created by crossing VE-cadherin cre mice^30^ with IL-7Rα^fl/fl^ mice (Jackson^31^ Laboratory, Bar Harbor, USA). A control mouse line was established from the same genetic background as IL-7Rα^ΔEC^ mice (i.e. VE-cadherin^wt/wt^ x IL-7Rα^fl/fl^ mice). The deletion efficiency in IL-7Rα^ΔEC^ mice was determined by quantitative PCR on LECs isolated from LNs, as described in the online Supplementary Methods. All mice were bread under specific pathogen free (SPF) conditions in the ETH Rodent Center HCI facility. Experiments were performed using groups of 8–13 weeks old, age- and sex-matched animals.

### Approval

All *in vivo* experiments in mice were approved by the Cantonal Veterinary Office Zurich (protocol numbers 268/2014 & 238/2017), and all methods were performed in accordance with the relevant guidelines and regulations.

### Cloning, expression, purification and activity of mIL-7-Fc

See Supplemental Methods.

### Oxazolone-induced inflammation in K14-VEGF-A mice

A CHS response was induced in K14-VEGF-A mice as described previously^4^. In brief, mice were anesthetized by inhalation of anesthesia (isoflurane, 2.5%, Piramal Enterprises, Mumbai, India). The belly was shaved with a razor. 2% oxazolone (4-ethoxymethylene-2-phenyl-2-oxazoline-5-1, Sigma-Aldrich) in acetone/olive oil (4/1 v:v) were applied to the belly (50 μl) and to each paw (5 μl). Mice were kept under anesthesia for approximately 20 minutes to ensure absorption of oxazolone by the skin. After 5 days mice were challenged with 10 μl 1% oxazolone in acetone/olive oil (4/1 vol:vol) on each side of the ear and the baseline ear thickness was determined using an ear caliper (Brütsch Rüegger, Urdorf, Switzerland). After 7 days mice were anesthetized with inhalation of anesthesia (isoflurane, 2.5%) and the ear thickness was measured. Mice were randomized into 3 treatment groups with equal average increase in ear thickness and similar standard deviation in each group. Mice were treated i.p. with 37 μg mIL-7-Fc or corresponding control fusion protein (KSF-Fc; non-specific single chain Fv antibody fragment against hen egg lysozyme^53^, fused to an IgG1 Fc fragment, and produced in our lab^37^, or 400 μg anti-IL-7α blocking antibody (clone A7R34, Bioexcell, West Lebanon, USA) or corresponding IgG control (clone 2A3, Bioexcell) every other day. I.p. treatment (100 μg) with dexamethasone (Mepha, Switzerland) was either given every day or every other day^54^, as indicated in the experimental layout in Figs 2a, 3a or 4a. Ear thickness was measured on all treatment days. Lymphatic drainage was assessed on day 14. Mice were sacrificed on day 15. Ears and dLN were harvested for further analysis by FACS or immunofluorescence.

### Imiquimod-induced inflammation

C57BL/6 mice were anesthetized by inhalation of anesthesia (isoflurane, 2.5%). The baseline ear thickness was measured using an ear caliper (Brütsch Rüegger, Urdorf, Switzerland). Imiquimod-containing cream (Aldara 5% (50 mg/g), Mepha, Switzerland) was applied with a spatula to the two sides of each ear (approximately 30 mg of cream per mouse). Cream was applied on day 1–5, 7, 9, 11, and 13 and the ear thickness was determined. In some cases, an additional dose of imiquimod was given on day 6. On day 7, when the peak in ear thickness was reached (±200 μm increase from baseline), mice were randomized into 3 treatment groups with an equal average increase in ear thickness and similar standard deviation in each group. Mice were treated i.p. with 400 μg anti-IL-7Rα blocking antibody (clone A7R34, Bioexcell) or corresponding IgG control (clone 2A3, Bioxcell), or 100 μg dexamethasone (Mepha) on days 7, 9, 11, and 13 after oxazolone challenge. Lymphatic drainage was assessed on day 14. Mice were sacrificed on day 15. Ears and dLNs were harvested for further analysis by FACS.

In some experiments the establishment of imiquimod-induced inflammation was studied in IL-7Rα^ΔEC^ mice and in the corresponding control mice (CTR: VE-cadherin^wt/wt^ x IL-7Rα^fl/fl^ mice). Lymphatic drainage was determined on day 7. Mice were sacrificed on day 8 and the ears and dLNs were harvested for analysis by FACS.

### Acute TPA-induced inflammation

IL-7R^αΔEC^ mice and the corresponding control mice were anesthetized by inhalation of anesthesia (isoflurane, 2.5%). The baseline ear thickness was measured using an ear caliper (Brütsch Rüegger, Urdorf, Switzerland) and 1 μg 12-O-tetradecanoylphorbol 13-acetate (TPA/PMA, Sigma-Aldrich) in acetone (Sigma-Aldrich) was applied to each side of the ear on day 1. Lymphatic drainage was measured on day 2 and mice were sacrificed on day 3. The ears and dLNs were harvested for analysis by FACS.

### FACS analysis of ear and LN single-cell suspensions

Different immune cell populations in ears, LNs, and spleen were analyzed by FACS. To this end single cell suspensions were created as previously described^55^. In brief, mice were sacrificed and the ears and LNs were harvested. Ears were split along the cartilage and cut in small pieces. Ears and LNs were digested in PBS containing 4 mg/ml collagenase type IV (Thermo Fisher) for 45 minutes at 37 °C. LNs and spleens that were used to determine leukocyte expansion after mIL-7-Fc treatment were not digested. Tissues were passed through a 40-μm cell strainer, centrifuged, resuspended in FACS buffer (PBS containing 2% FCS (Thermo Fisher) and 2 mM EDTA (Sigma-Aldrich)), and filtered through a 40-μm filter tube. Splenocytes were subjected to red blood cell lysis after straining by incubation with ACK buffer containing 150 mM ammonium chloride (Sigma-Aldrich), 10 mM potassium bicarbonate (Sigma-Aldrich), and 0.1 mM EDTA-Na_2_ (Sigma-Aldrich) for 5 minutes on ice. AccuCheck counting beads (Thermo Fisher) or Flow-Count Fluorospheres (Beckman Coulter, Brea, USA) were added to the samples after cell straining to enable absolute cell quantification. Samples were stained with antibodies in FACS buffer for 20 minutes on ice using the following anti-mouse antibodies (all from Biolegend, unless noted): CD45-PerCP, CD45-APC/Cy7 or CD45-PE/Cy7 (clone 30F-11), CD4-PE, CD4-FITC or CD4-APC (clone GK1.5), CD8a-FITC (clone 53-6.7), CD3-APC (clone 145-2C11), TCRβ-PE/Cy7 or TCRβ-Brilliant Violet 421 (clone H57-597), CD44-FITC or CD44-APC (clone IM7), Ly6G-PerCP/Cy5.5 or Ly6G-PE (clone 1A8), CD11c-APC or CD11c-PE/Cy7 (clone N418), CD11b-FITC (clone M1/70), MHCII-PE or MHCII-BV421 (clone M5/114.15.2), podoplanin-APC (clone 8.1.1), CD31-FITC (clone MEC 13.3, from BD Biosciences), CD127-PE (clone SB/199) and the corresponding isotype control (rat IgG2b-PE, clone RTK4530). In all experiments an Fc receptor blocking step with anti-mouse CD16/32 (clone 93) was performed prior to FACS staining, and a live/dead cell staining with zombie-NIR or zombie-Aqua (both from Biolegend) was performed prior to recording. All FACS samples were acquired on a FACS Canto (BD Bioscience) using FACSDiva software (BD Bioscience) or on a Cytoflex (Beckman Coulter) using CytExpert Software (Beckman Coulter). Data were analyzed using FlowJo software (Treestar). All other FACS analyses are described in the online Supplemental Methods.

### Protein extraction from ear lysates

Harvested ears were split along the cartilage and cut into small pieces. 500 μl lysis buffer (10 ml, containing 50 mM Tris, 150 mM NaCl (Sigma-Aldrich), and a cOmplete Mini EDTA-free Protease Inhibitor Cocktail tablet (Sigma-Aldrich)) and a stainless steel metal bead (Qiagen, Hombrechtikon, Switzerland) were added to the ear pieces. Tissues were homogenized 4 times for 60 seconds at maximum speed using a Tissue Lyser (Qiagen). Subsequently, tissues were centrifuged, the supernatant was collected, and sent for IL-7 and TSLP determination to Cytolab for analysis with a multiplexed particle-based cytometric cytokine assay (Regensdorf, Switzerland).

### Lymphatic drainage assay

The lymphatic drainage assay was performed as previously described^29,56^. In brief, mice were anesthetized by inhalation of anesthesia (isoflurane, 2.5%) and the base of the ears were shaved with a razor. Three μl P20D800 (3 μM- synthesized as described in the online Supplemental Methods) were injected i.d. into the ear skin using a 29G insulin syringe (Terumo,Tokyo, Japan). Subsequently, the mouse was placed into an IVIS imaging system (Perkin Elmer, Waltham, MA) with the ears taped down flat and a fluorescence image (λ_ex_ = 745 nm, λ_em_ = 800 nm, exposure time 4 seconds, binning 2) was acquired. Imaging was repeated 1, 2, 4, 6, and 24 hours after injection. Mice were housed in their cages in between imaging time points and anesthetized by inhalation of isoflurane for approximately 5 minutes for each imaging session. For the analysis, regions of interest (ROI) were drawn around the ears and the average fluorescence intensity was measured in each ROI using Living Image 4.0 software (Perkin Elmer). After subtracting the background fluorescence intensity of uninjected ears, the average fluorescence intensities in each ROI were normalized to the initial amount of dye injected (average fluorescence intensity at time 0). The normalized average fluorescence intensities were plotted against time and fitted to a one-phase exponential decay model from which the tracer clearance half-life was determined.

### Immunofluorescence staining and analysis of ear sections

See Supplemental Methods.

### Statistical analysis

Graphs were generated in GraphPad prism 7 (La Jolla, USA) and statistical analysis was performed using the same software. The student t-test (unpaired, two-tailed) was used when comparing two groups. One-way ANOVA with a Tukey-post hoc test was used to compare three or more groups. For comparison of more than two groups in repeated measurements (i.e. ear thickness measurements) a 2-way ANOVA with Bonferroni correction was used. Differences were considered statistically significant when p < 0.05. Mean and standard error of the mean (SEM) are shown.

## Supplementary information

Supplementary Methods

supplemental Dataset

## Data Availability

The datasets generated during and/or analysed during the current study are available from the corresponding author on reasonable request.
