# Supplementary material for: Opposing roles of endothelial and leukocyte-expressed IL-7Rα in the regulation of psoriasis-like skin inflammation
Source: Sci Rep. 2019 Aug 12;9:11714. doi: 10.1038/s41598-019-48046-y (PMC6691132; doi:10.1038/s41598-019-48046-y)
Supplement: Supplementary file 1 — Supplementary Methods [file 41598_2019_48046_MOESM1_ESM.pdf]

Opposing roles of endothelial and leukocyte-expressed IL-7Rα in the regulation of psoriasis-like skin inflammation

Martina Vranova[1], Mona C. Friess[1], Neda Haghayegh Jahromi[1#], Victor Collado-Diaz[1#], Angela Vallone[1], Olivia Hagedorn[1], Maria Jadhav[1], Ann-Helen Willrodt[1], Anna Polomska[1], Jean-Christophe Leroux[1], Steven T. Proulx[1], Cornelia Halin[1*]

[1] Institute of Pharmaceutical Sciences, ETH Zurich, Switzerland

[#] These authors contributed equally to this work

***Correspondence:**
Corresponding Author
cornelia.halin@pharma.ethz.ch

Supplementary Methods

LN LEC isolation and purification

LNs were harvested from IL-7Rα$^{\Delta EC}$ and WT mice and digested for one hour at 37°C in RPMI medium (Sigma-Aldrich, St. Louis, USA) containing 0.25 mg/ml Liberase TL (Roche, Basel, Switzerland) and 200’000 units/ml DNase I (Roche). LNs were cut in pieces with microscissors and pipetted repeatedly during the digestion process to disintegrate the tissue. In a next step, LN digests were passed through a 70 µm strainer. Lymphocytes were resuspended in αMEM medium containing 10% fetal calf serum (FCS), 1% antibiotic antimycotic (Anti Anti), 1% L-glutamine (all from Thermo Fisher, Waltham, USA). Cells were seeded in cell culture plates coated with 10 ug/ml human plasma fibronectin (Millipore, Billerica, USA) and 10 µg/ml collagen type I (Advanced BioMatrix, San Diego, USA) and cultured at 37 °C in the presence of 5% CO_2 and 100% relative humidity. After 6 days of culture, cells were detached with accutase (Sigma-Aldrich) and LECs were purified using CD31 microbeads (Miltenyi Biotec, Cologne, Germany).

Genotyping PCR

DNA from cultured LN LECs was extracted using the NucleoSpin Tissue kit (Machery-Nagel, Düren, Germany). PCRs were performed to detect cre-mediated recombination, as well as the WT sequence. The forward primer 5’ GGT ACC TTG AGC TCA GAA ATT GG 3' (primer 1) or 5’ ACA GTG GGG CTC TTT TAC GA 3’ (primer 2) respectively was used with the reverse primers 5’GAG GTA GAT GGC CTG CTT TAG 3’ (primer 3). A quantitative real-time PCR was performed using the primer pair for the WT sequence on a Fast Real-time PCR system (Applied Biosystems, Thermo Fisher).

Cloning of mIL-7-Fc

Murine IL-7 cDNA was amplified from a murine IL-7-containing pMD18 plasmid (Sino Biological Inc., Beijing, China) by PCR. To optimize expression of the mIL-7-Fc protein, the native secretion sequence was replaced by a secretion sequence more suitable for production in CHO-S cells (kindly provided by Dario Neri, ETH Zurich [1]). In a first PCR we appended the first part of the secretion sequence to the N terminus with the forward primer: 5’ TCC TCC TGT TCC TCG TCG CTG TGG CTA CAG GTG TGC ACT CGG AGT GCC ACA TTA AAG ACA AAG AAG GTA 3’. The backward primer appended the coding sequence for the first 6 amino acids of the hinge region, that links mIL-7 to the murine Fc: 5’ CTT ACA ACC ACA ATC CCT GGG CAC TAT ACT GCC CTT CAA AAT TTT ATT CCA ACA AGT TTT TAT TTC TC 3’. In a second PCR we added the first part of the secretion sequence and a HindIII restriction site

with the following primer set: 5' CCC AAG CTT GTC GAC CAT GGG CTG GAG CCT GAT CCT CCT GTT CCT CGT CGC TGT GGC 3' and 5' CTT ACA ACC ACA ATC CCT GGG CAC TAT ACT GCC CTT CAA AAT TTT ATT CCA ACA AGT TTT TAT TTC TC 3'. The Fc fragment (C_{H2} and C_{H3} domains of murine IgG1) was amplified from a commercial cDNA (Source BioScience, Berlin, Germany). The following primer set was used: 5' GTG CCC AGG GAT TGT GGT TGT AAG CCT TGC ATA TGT ACA GTC CCA GAA G 3'and 5' TTT TCC TTT TGC GGC CGC TCA TTA AGC TAT TTA CCA GGA GAG TGG GAG AGG 3'. The sequences for mIL-7 and Fc were assembled in a PCR with following primers: 5' CCC AAG CTT GTC GAC CAT GGG CTG GAG CCT GAT CCT CCT GTT CCT CGT CGC TGT GGC 3' and 5' TTT TCC TTT TGC GGC CGC TCA TTA AGC TAT TTA CCA GGA GAG TGG GAG AGG 3'. All PCRs were carried out with the OneTaq polymerase mix (New England Biolabs, Ipswich, USA). The assembled sequence for mIL-7-Fc was digested with HINDIII and NOTI restriction enzymes (both from New England Biolabs) and gel-purified, and ligated into a HINDIII/NOTI digested pcDNA3.1 plasmid (Thermo Fisher, Waltham, USA). PCR, digestion, and ligation products were purified with a PCR cleanup and gel extraction kit (Machery-Nagel). The plasmid containing the mIL-7-Fc sequence was transformed into TOP10 E. Coli (Thermo Fisher) by heat shock (30 minutes on ice, 30 seconds at 42°C, 2 minutes on ice) and single colonies were screened for the correct sequence. The plasmid containing the correct sequence was purified with a plasmid DNA purification kit (Machery-Nagel).

Expression and purification of mIL-7-Fc

Expression and purification of mIL-7-Fc in CHO cells was performed as described previously [2]. In brief, CHO cells were transfected with plasmid containing mIL-7-Fc by Polyethylenimine-mediated transient gene expression. CHO cells were incubated in PRO-CHO4 medium (Lonza, Basel, Switzerland) containing 2mM glutamine (Thermo Fisher), hydroxytryptamine (Thermo Fisher) and antibiotic antimycotic (Anti Anti, Thermo Fisher) for one week at 31°C in a shaking incubator at 120 rotations per minute. The protein was purified by running the CHO cell supernatant over a PD10 resin (GE Healthcare, Chicago, USA) followed by a protein A resin (Sino Biological). After 2 washing steps in buffer containing 100 mM NaCl (Sigma-Aldrich), 5 mM EDTA (Sigma-Aldrich), 1% polysorbate 20 (Sigma-Aldrich) and 100 mM NaCl (Sigma-Aldrich), 5 mM EDTA (Sigma-Aldrich) respectively, the protein was eluted from the protein A resin with 0.1 M triethylamine PH 9 (Sigma-Aldrich) and the PH was adjusted immediately after elution by addition of 1/10 of the volume of 1M Tris-HCl (PH 7). The protein was dialyzed overnight in PBS and sterile filtered through a 0.22 μm PES filter (TPP, Trasadingen, Switzerland). The concentration was determined by measuring the absorption at 280 nm on a

Nanodrop Spectrophotometer (Witec, Ulm, Germany). The protein was shock-frozen in liquid nitrogen and stored at -80°C. The correct size of the protein (unglycosylated monomer is 41 kDa) was verified by size exclusion chromatography on a Superdex 200 5/150 GL column (GE Healthcare Life Sciences) and by SDS-PAGE.

in vitro T cell survival assay

LNs, isolated from C57BL/6 mice, were passed through a 40 µm cell strainer, centrifuged, and suspended in RPMI medium (Sigma-Aldrich), supplemented with 2% FCS (Thermo Fisher), 2 mM glutamine (Thermo Fisher), 4 mM Sodium Pyruvate (Thermo Fisher), 15 mM HEPES (Thermo Fisher), 0.09% 2-mercaptoethanol (Sigma-Aldrich). 200 000 cells were incubated with 50 ng/ml, 10 ng/ml, 5 ng/ml, 1 ng/ml, 0.5 ng/ml, 0.1 ng/ml, and 0.01 ng/ml human IL-7, murine IL-7, or the molar equivalent of mIL-7-Fc for 3 days at 37°C. Cells were stained with primary antibodies in FACS buffer (PBS containing 2 % FCS (Thermo Fisher) and 2 mM EDTA (Sigma-Aldrich)) for 20 minutes on ice. Following primary anti-mouse antibodies (Biolegend, San Diego, USA) were used: CD45-APC (clone 30-F11), CD4-PE (clone GK1.5), CD8a-FITC (clone 53-6.7). Samples were acquired on a FACS Canto (BD Bioscience, Franklin Lake, USA) using FACSDiva software (BD Bioscience). Data were analyzed using FlowJo software (Treestar, Ashland, USA).

in vivo T cell expansion by mIL-7-Fc

C57BL/6 mice were treated intraperitoneally (i.p.) with PBS, 1.5 µg/15 µg human IL-7/anti-IL-7 antibody (clone M25) complexes [3], and the 1, 5 and 10-fold molar equivalents of mIL-7-Fc every second day for one week. Mice were sacrificed on day 8. Brachial LNs, inguinal LNs, and spleen were harvested, passed through a 40-µm cell strainer, centrifuged, resuspended in FACS buffer (PBS containing 2 % FCS (Thermo Fisher) and 2 mM EDTA (Sigma-Aldrich)), and filtered through a 40-µm filter tube. Splenocytes were subjected to red blood cell lysis after straining by incubation with ACK buffer containing 150 mM ammonium chloride (Sigma-Aldrich), 10 mM potassium bicarbonate (Sigma-Aldrich), and 0.1 mM EDTA-Na_2 (Sigma-Aldrich) for 5 minutes on ice. Accucheck counting beads (Thermo Fisher) or Flow-Count Fluorospheres (Beckman Coulter, Brea, USA) were added to the samples after cell straining to enable absolute cell quantification. Samples were stained with antibodies in FACS buffer for 20 minutes on ice with CD45-PerCP (clone 30F-11) for subsequent FACS analysis.

Human LEC cell culture

Human primary LECs isolated from neonatal human foreskins were kindly provided by Dr. Michael Detmar [4]. Cells were cultured on plates pre-coated with collagen type I (Advanced BioMatrix, Poway,USA; 3,1 mg/ mL) and/or fibronectin (Millipore, Billerica, USA; 1mg/mL) in Endothelial Basal Medium (EBM; Lonza, Walkersville, MD, USA) supplemented with 20% fetal bovine serum (FBS) (GIBCO, Paisley, UK), antibiotic antimycotic solution (1x; Fluka, Buchs, Switzerland), L-glutamine (2 mM; Fluka), hydrocortisone (10 µg/ml; Fluka) and N^6,2′-O-dibutyryladenosine 3′,5′-cyclic monophosphate sodium salt (cAMP, 2.5 x 10^{-2} mg/ml; Fluka).

Proliferation Assay

2000 LECs/well or 1500 BECs/well were seeded into collagen and/or fibronectin-coated 96-well plates (8 -12 wells per condition) and treated with different concentrations of human TSLP (from 0.5 to 50 ng/ml, PeproTech) or human VEGF-A (20ng/ml, PeproTech). After 72 hours, cells were incubated with 5-methylumbelliferylheptanoate (MUH, Sigma-Aldrich, Steinheim, Germany) and viable cells quantified spectrophotometrically using a SpectraMax Gemini EM (Bucher Biotec AG, Basel, Switzerland), as previously described [5].

Scratch-wound assay

LECs were grown to confluence in collagen/fibronectin coated 24-well plates and incubated for 24 hours in starvation medium (EBM, supplemented with 2% FCS and antibiotic-antimycotic solution). Two cross-shaped scratches were made in each well, using a sterile 200-µL pipette tip. Monolayers were washed twice with PBS, and 500 µL of starvation medium supplemented with TSLP or VEGF-A. Pictures of crosses in the monolayers (cell-free zones) were taken immediately after scratching and 16-24 hours later. For each cross (8 - 10 per condition) the percentage of the surface area closed after 16-24 hours was calculated using T-scratch software[6].

FACS of IL-7Ra and TSLPR expression in murine LECs *in vivo* and human LECs *in vitro*

Single cell suspensions were created as previously described [7]. In brief, mice were sacrificed and the ears were harvested. Ears were split along the cartilage and cut in small pieces. Ears were digested in PBS containing 4 mg/ml collagenase type IV (Thermo Fisher) for 45 minutes at 37°C. Tissues were passed through a 40-µm cell strainer, centrifuged, resuspended in FACS buffer (PBS containing 2 % FCS (Thermo Fisher) and 2 mM EDTA (Sigma-Aldrich)), and filtered through a 40-µm filter tube. Samples were stained with antibodies in FACS buffer for 20 minutes on ice using the following anti-mouse antibodies (all from Biolegend, unless noted): CD45-PerCP (clone 30F-11), CD31-APC (clone MEC13.3), podoplanin-PE/Cy7 (clone 8.1.1), IL-7Rα-

FITC (clone SB/199), TSLPR-Fluorescein (polyclonal goat, R&D Systems) and zombie-NIR (live/dead staining). For FACS analysis of cultured human LECs, cells were detached using accutase (Sigma-Aldrich), re-suspended in FACS buffer and stained with anti-human IL-7Rα-Alexa Fluor 488 (clone A019D5) and anti-human TSLPR-APC (clone 1B4) or the corresponding isotype controls (all from BioLegend). For intracellular staining of TSLPR on human LECs, cells were fixed with 4% paraformaldehyde (Sigma-Aldrich) and subsequently stained in FACS buffer containing 0.3% saponin (Sigma-Aldrich) with goat anti-human TSLPR or corresponding isotype control (both R&D Systems), followed by anti-goat PE (Caltag Laboratories).

Synthesis of P20D800

P20D800 was synthesized as described previously [8]. In brief, 300 nmol methoxypoly(ethylene glycol) (referred to as PEG-20) (20 kDa, JenKem Technologies, Plano, USA) were dissolved in 400 µl anhydrous DMSO (Sigma-Aldrich) at 37 °C. Subsequently, PEG-20 was labeled with 300 nmol IRDye® 800CW NHS Ester (referred to as IRdye800) (LI-COR Biosciences, Lincoln, USA) for 6 hours at room temperature (RT) at 800 rotations per minute. The reaction mixture was diluted in 6 ml ultra-pure water and freeze-dried overnight. The next day the dry powder was re-dissolved in 300 µL HEPES buffered saline (HEPES 20 mmol/L, NaCl 142 mmol/L, pH 7.4, Sigma-Aldrich) and purified over 2 Fluorescent Dye Removal Columns (Thermo Fisher). The concentration was determined by measuring the absorption at 778 nm in methanol on a Cary 300 Bio UV-spectrophotometer (Agilent Technologies, Santa Clara, USA).

Immunofluorescence staining of ear sections

Ears were embedded in OCT compound (Richard-Allan Scientific, San Diego, USA) and frozen on liquid nitrogen. Ears were cut along the midline and re-embedded in OCT compound to allow longitudinal sectioning. 6 µm sections were cut on a CryoStar NX50 (Thermo Fisher). For immunofluorescence staining sections were fixed for 2 minutes at -20°C in acetone (Sigma-Aldrich) and 5 minutes at 4°C in 80% methanol. Subsequently, the sections were washed in Tris-buffered saline for 3 x 2 minutes, blocked with 12% BSA (Sigma-Aldrich) in PBS for 1 hour at RT and stained with the primary antibody in antibody diluent (Zytomed systems, Berlin, Germany) for 1 hour at RT. After a washing step (3 x 2 minutes) in TBS, secondary antibodies were added for 1 hour at RT. Cell nuclei were stained with DAPI (Thermo Fisher) and the samples were washed again in Tris-buffered saline (3 x 2 minutes) before mounting with Mowiol (Calbiochem, Darmstad, Germany). The following primary anti-mouse antibodies were used: rat MECA-32 (Biolegend), rabbit von Willebrand factor (polyclonal, Dako Agilent, Santa Clara, USA), rabbit LYVE-1 (polyclonal, AngioBio, San Diego, USA), rabbit keratin 6 (Covance, Princeton, USA), rabbit keratin 10 (Covance). The following secondary antibodies were used:

donkey anti-rat Alexa-594, donkey anti-rabbit Alexa-488, donkey anti-rabbit Alexa-594 (all from Thermo fisher). Pictures were acquired of the thicker ear half, between the cartilage and epidermis on an Axioskop 2 mot plus microscope (Carl Zeiss. Okerbochen, Germany) using an AxioCam MRm camera and a Plan-NEOFLUAR 10x/0,30 44 03 30 objective (Carl Zeiss AG).

Morphometric vessel analysis of ear sections

FIJI software was used to analyze the vessel area and number. A ROI was drawn around the thicker ear half between the cartilage and the epidermis and along the epidermal basement membrane. After thresholding and smoothening the image, vessel area and number, and keratin area were determined using the particle analyzer or area measurement tool respectively in FIJI. 3-5 images were analyzed per mouse. The experimenter who analyzed the images was blinded.

References

1 Zuberbuhler, K. *et al.* A general method for the selection of high-level scFv and IgG antibody expression by stably transfected mammalian cells. *Protein Eng Des Sel* **22**, 169-174, doi:10.1093/protein/gzn068 (2009).

2 Casi, G., Huguenin-Dezot, N., Zuberbuhler, K., Scheuermann, J. & Neri, D. Site-specific traceless coupling of potent cytotoxic drugs to recombinant antibodies for pharmacodelivery. *J Am Chem Soc* **134**, 5887-5892, doi:10.1021/ja211589m (2012).

3 Boyman, O., Ramsey, C., Kim, D. M., Sprent, J. & Surh, C. D. IL-7/anti-IL-7 mAb complexes restore T cell development and induce homeostatic T Cell expansion without lymphopenia. *J Immunol* **180**, 7265-7275 (2008).

4 Hirakawa, S. *et al.* Identification of vascular lineage-specific genes by transcriptional profiling of isolated blood vascular and lymphatic endothelial cells. *Am J Pathol* **162**, 575-586, doi:10.1016/S0002-9440(10)63851-5 (2003).

5 Detmar, M., Tenorio, S., Hettmannsperger, U., Ruszczak, Z. & Orfanos, C. E. Cytokine regulation of proliferation and ICAM-1 expression of human dermal microvascular endothelial cells in vitro. *J Invest Dermatol* **98**, 147-153 (1992).

6 Geback, T., Schulz, M. M., Koumoutsakos, P. & Detmar, M. TScratch: a novel and simple software tool for automated analysis of monolayer wound healing assays. *Biotechniques* **46**, 265-274 (2009).

7 Halin, C., Tobler, N. E., Vigl, B., Brown, L. F. & Detmar, M. VEGF-A produced by chronically inflamed tissue induces lymphangiogenesis in draining lymph nodes. *Blood* **110**, 3158-3167, doi:10.1182/blood-2007-01-066811 (2007).

8 Proulx, S. T. *et al.* Non-invasive dynamic near-infrared imaging and quantification of vascular leakage in vivo. *Angiogenesis* **16**, 525-540, doi:10.1007/s10456-013-9332-2 (2013).
