# Supplementary material for: Opposing roles of endothelial and leukocyte-expressed IL-7Rα in the regulation of psoriasis-like skin inflammation
Source: Sci Rep. 2019 Aug 12;9:11714. doi: 10.1038/s41598-019-48046-y (PMC6691132; doi:10.1038/s41598-019-48046-y)
Supplement: Supplementary file 2 — supplemental Dataset [file 41598_2019_48046_MOESM2_ESM.pdf]

Opposing roles of endothelial and leukocyte-expressed IL-7Rα in the regulation of psoriasis-like skin inflammation

Martina Vranova[1], Mona C. Friess[1], Neda Haghayegh Jahromi[1#], Victor Collado-Diaz[1#], Angela Vallone[1], Olivia Hagedorn[1], Maria Jadhav[1], Ann-Helen Willrodt[1], Anna Polomska[1], Jean-Christophe Leroux[1], Steven T. Proulx[1], Cornelia Halin[1*]

[1] Institute of Pharmaceutical Sciences, ETH Zurich, Switzerland

[#] These authors contributed equally to this work

***Correspondence:**
Corresponding Author
cornelia.halin@pharma.ethz.ch

Supplemental Figures and Figure Legends

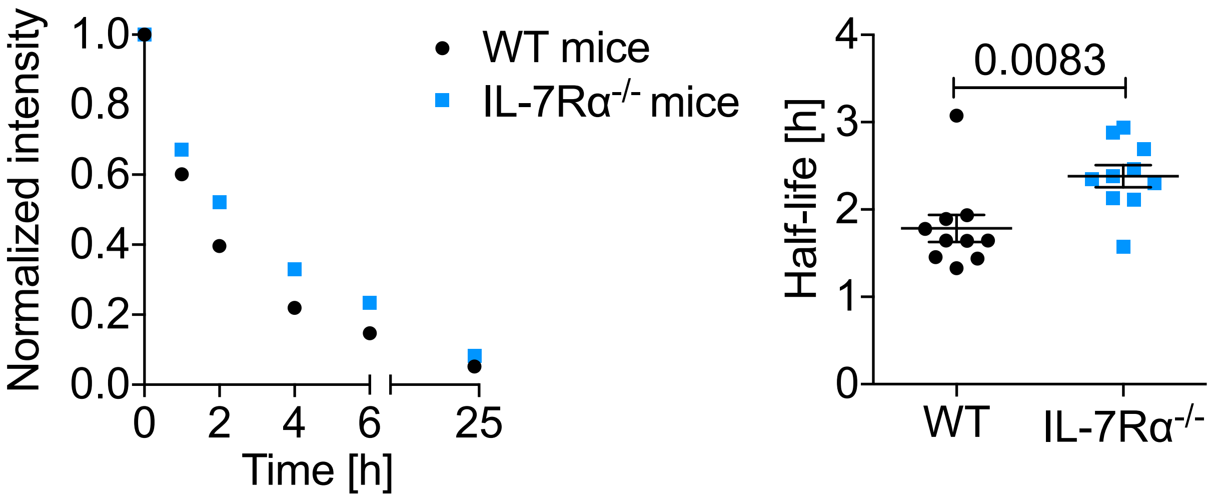

Suppl. Fig. 1. Global deletion of IL-7Rα decreases lymphatic drainage. Mice were injected with a P20D800 conjugate i.d. into the ear skin. Clearance of the tracer was monitored over 24 hours using an IVIS imaging system. Average clearance plots of P20D800 (left) and calculated half-lives (right) in WT and IL-7Rα$^{-/-}$ mice (pooled data from 2 similar experiments with a total of 10 mice per group).

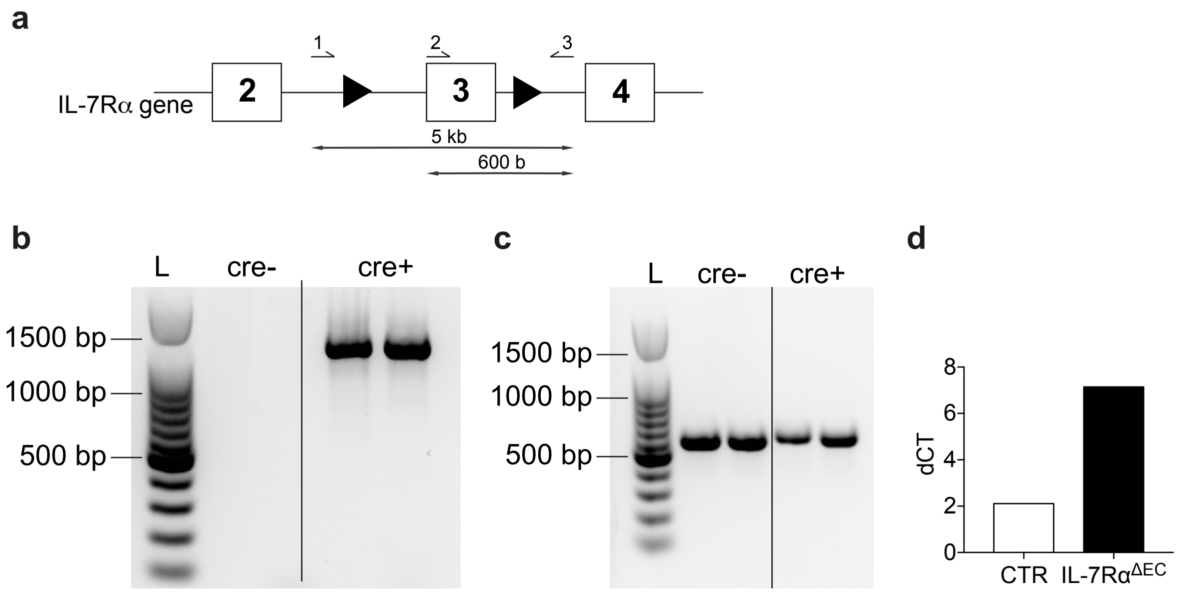

Suppl. Fig. 2. Confirmation of IL-7Rα gene deletion in LN LECs of IL-7Rα$^{\Delta EC}$ mice. (**a**) Schematic representation of exons 2-4 (boxes) of the IL-7Rα gene and the position of the loxP sequences (triangles). LN LECs were isolated from VE-cadherin$^{cre/cre}$ x IL-7Rα$^{fl/fl}$ (cre^{+}, IL-7Rα$^{\Delta EC}$) and VE-cadherin$^{wt/wt}$ x IL-7Rα$^{fl/fl}$ (cre^{-}) control mice. PCR was performed on DNA from cre^{+} and cre^{-} mice with the indicated primers. (**b**) PCR performed with primers 1 and 3 only amplifies the sequence when cre-mediated recombination of the IL-7Rα exon 3 has taken place. Thus, bands can only be detected in cre^{+} mice. (**c**) PCR performed with primers 2 and 3 from scheme in (a) only amplifies the sequence if cre-mediated recombination has not taken place. Bands can be detected in cre^{-} control mice and to a lesser extent also in cre^{+} mice, indicating that the cre-mediated deletion of exon 3 of the IL-7Rα is not complete. Representative gel images from three independent experiments are shown in (**b, c**). (**d**) Quantitative real-time PCR performed with primers 2 and 3. dCt (Ct value for primers 2 and 3 – Ct value for the housekeeping gene RpLp0) indicate an approximate deletion of the IL-7Rα of 97% in cre^{+} IL-7Rα$^{\Delta EC}$ compared to cre^{-} control (CTR) mice. Pooled data from two similar experiments are shown.

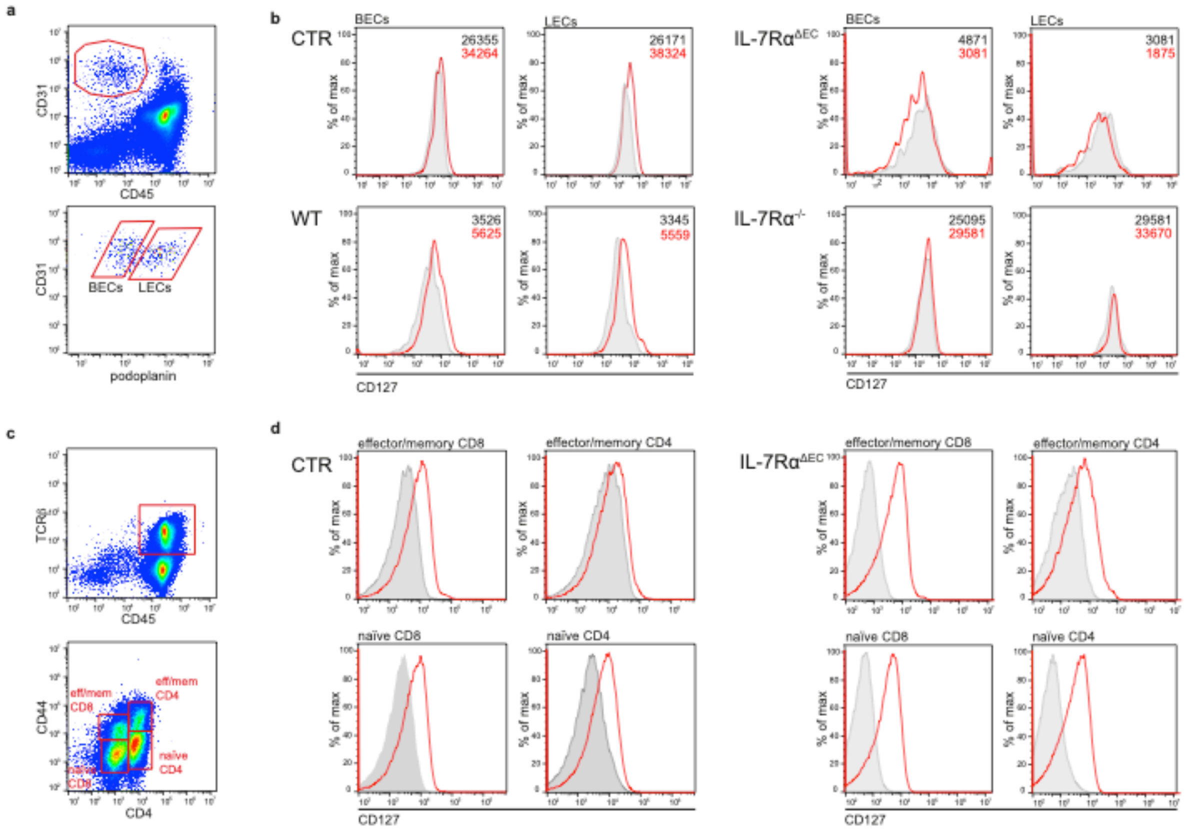

Suppl. Fig. 3. Analysis of IL-7Rα (CD127) protein levels on T cells and endothelial cells of WT, IL-7Rα$^{\Delta EC}$ and IL-7Rα$^{-/-}$ mice. Single-cell suspensions were generated from LNs of wild-type (WT), IL-7Rα$^{\Delta EC}$ (VE-cadherin$^{cre/cre}$ x IL-7Rα$^{fl/fl}$), control (CTR: VE-cadherin$^{wt/wt}$ x IL-7Rα$^{fl/fl}$) and IL-7Rα$^{-/-}$ mice, and IL-7Rα protein levels in T lymphocytes and endothelial cells were analyzed by FACS. (**a**) Depiction of the gating scheme used to identify BECs (CD45^{-}CD31^{+}podoplanin^{-}) and LECs (CD45-CD31^{+}podoplanin^{+}). (**b**) Expression of IL-7Rα protein in BECs and LECs of LN of the 4 indicated genotypes. The red lines indicate IL-7Rα expression, tinted histograms show the corresponding isotype control staining. (**c**) Depiction of the gating scheme used to identify naïve CD4^{+} T cells (CD45^{+}CD3^{+}CD4^{+}CD44^{low}), naïve CD8^{+} T cells (CD45^{+}CD3^{+}CD4^{-}CD44^{low}), CD4^{+} effector/memory (CD45^{+}CD3^{+}CD4^{+}CD44^{hi}) and CD8^{+} effector/memory cells (CD45^{+}CD3^{+}CD4^{-}CD44^{hi}) in LNs. (**d**) Expression of IL-7Rα protein in naïve and memory CD4^{+} and CD8^{+} T cells in LNs of IL-7Rα$^{\Delta EC}$ and control (CTR) mice. The red lines indicate IL-7Rα expression, tinted histograms show the corresponding isotype control staining. The corresponding mean fluorescence intensity (MFI) is written into each plot in colour (red: IL-7Rα, black: isotype control).

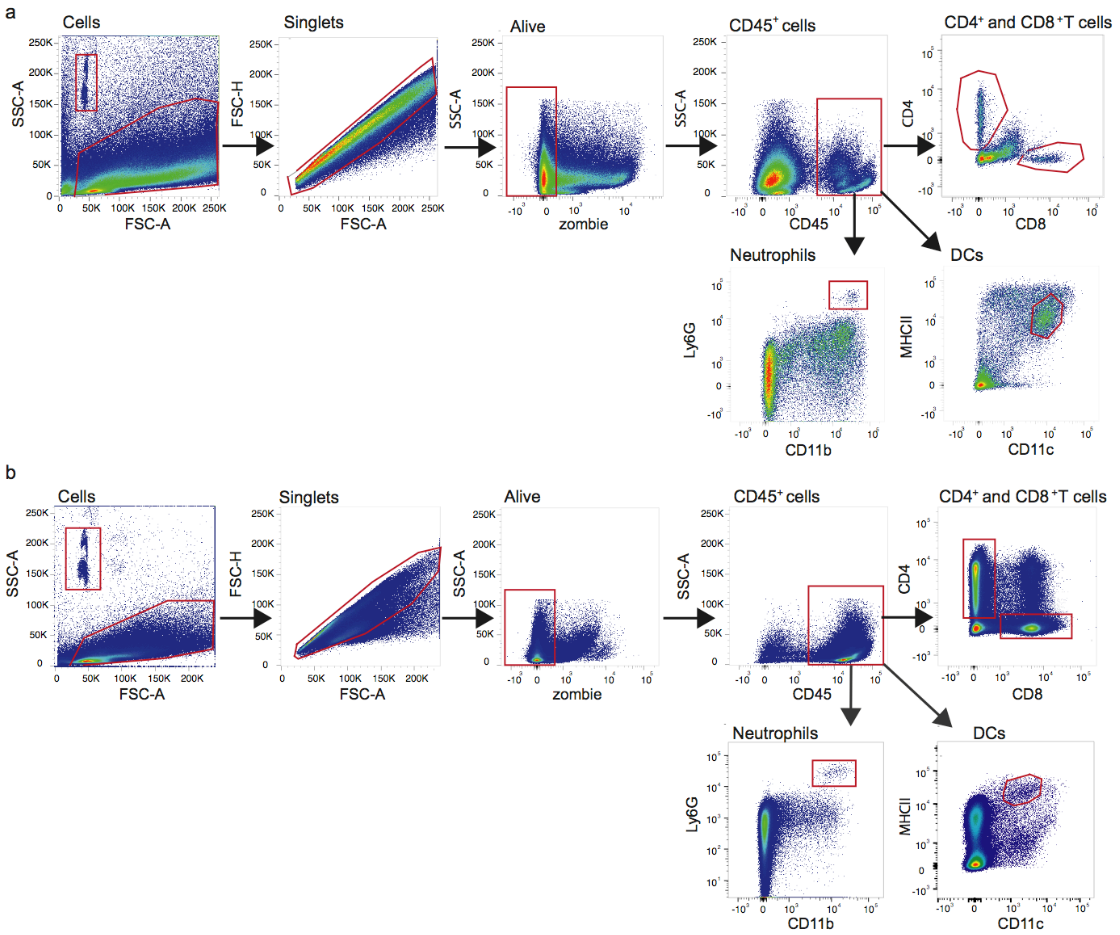

Suppl. Fig. 4. Gating strategy for immune cell populations by FACS in ear skin and LNs. Cells are selected on a side scatter (SSC) area versus forward scatter (FSC) area plot. Subsequently, singlets are gated on a FSC height (H) *versus* FSC-A plot, followed by the selection of live (zombie-negative) cells from which different CD45^{+} immune cell populations are gated and quantified in **(a)** ear skin and **(b)** LNs.

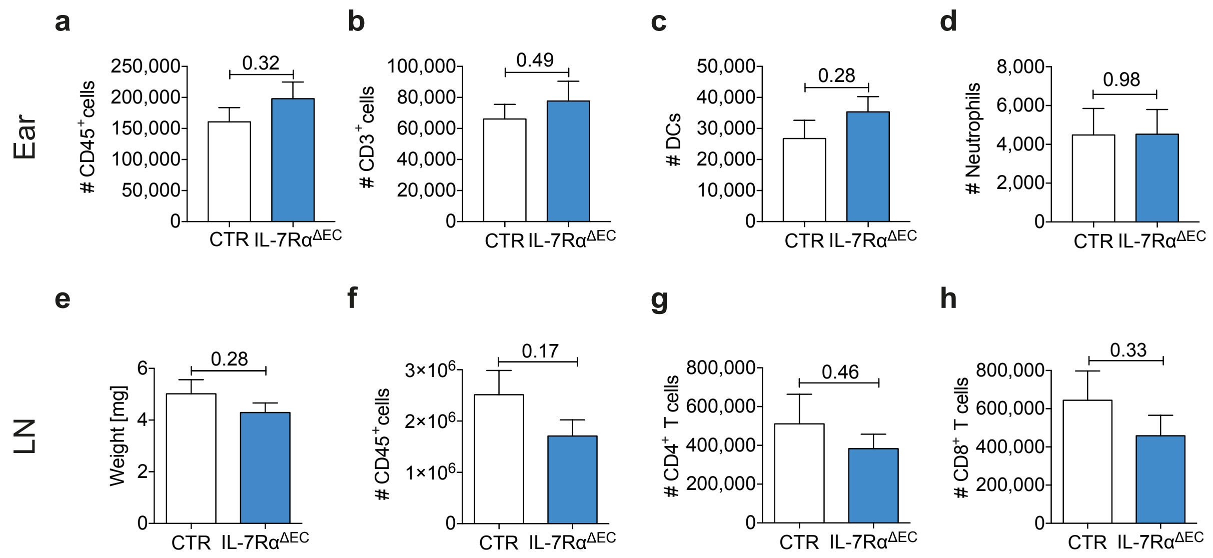

Suppl. Fig. 5. No marked differences in immune cell populations in steady state ear skin and LNs of IL-7Rα$^{\Delta EC}$ and control (CTR) mice. FACS-based quantification of the number of (**a, f**) CD45^{+} cells, (**b**) CD3^{+} T cells, (**c**) DCs, (**d**) neutrophils, (**g**) CD4^{+} T cells, (**h**) CD8^{+} T cells in (**a-d**) the ear skin and (**f-h**) inguinal LNs. (**e**) Inguinal LN weight. Both ears and inguinal LNs were pooled for FACS analysis. Pooled data (mean ± SEM) from 2 similar experiments with a total of 6-7 mice per group (**a-d**) and a total of 11 mice per group (**e-h**) are shown.

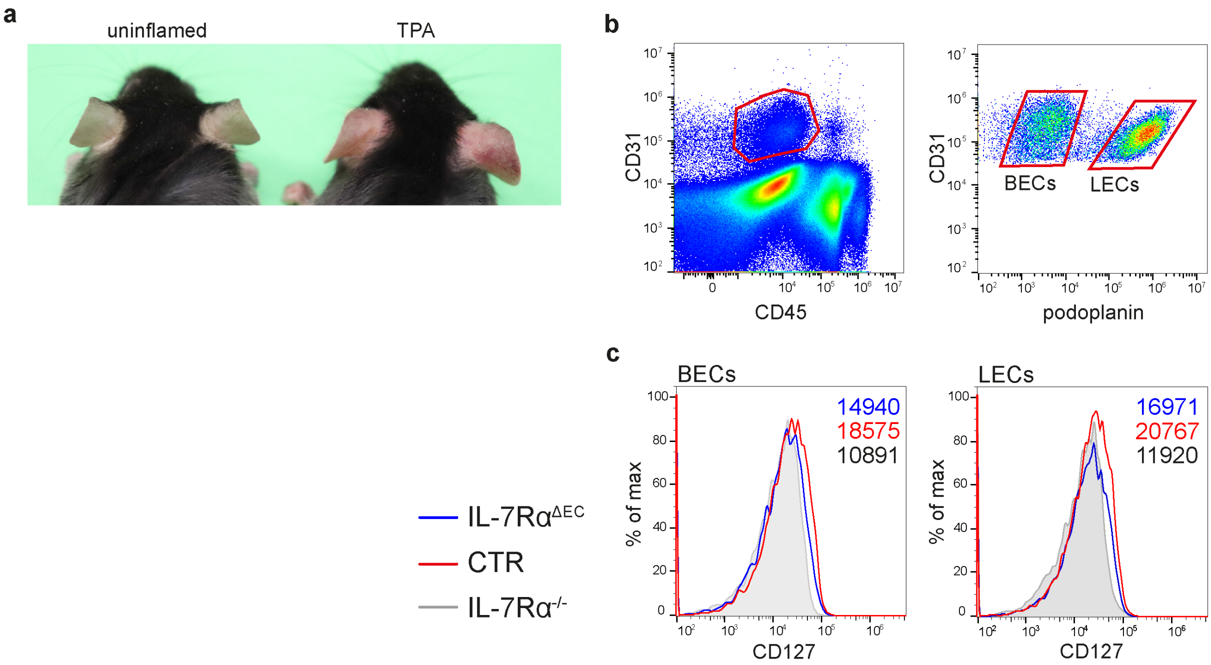

Suppl. Fig. 6. Characterization of IL7-Rα (CD127) expression in endothelial cells one day after TPA application. TPA was applied to the ears of WT mice. (**a**) Representative image of mice with uninflamed control ears (left) or with inflamed ears 1 day after TPA application (TPA - right). (**b,c**) FACS analysis was performed on TPA-inflamed ears (day 2 – see Fig. 1b) of IL-7Rα$^{\Delta EC}$ mice (VE-cadherin$^{cre/cre}$ x IL-7Rα$^{fl/fl}$), control (CTR) mice (VE-cadherin$^{wt/wt}$ x IL-7Rα$^{fl/fl}$) and IL-7Rα$^{-/-}$ mice, and CD127 (IL-7Rα) expression levels in endothelial cells was analyzed. (**b**) Depiction of the gating scheme used to identify dermal BECs (CD45^{-}CD31^{+}podoplanin^{-}) and LECs (CD45^{-}CD31^{+}podoplanin^{+}). (**c**) Expression of IL-7Rα protein in dermal BECs and LECs of the three different genotypes. The coloured lines indicate IL-7Rα expression in IL-7Rα$^{\Delta EC}$ (blue) and CTR (red), tinted histograms show the corresponding staining in IL-7Rα$^{-/-}$ mice. MFIs are written into each plot in colour (red: CTR, blue: IL-7Rα$^{\Delta EC}$, black: IL-7Rα$^{-/-}$).

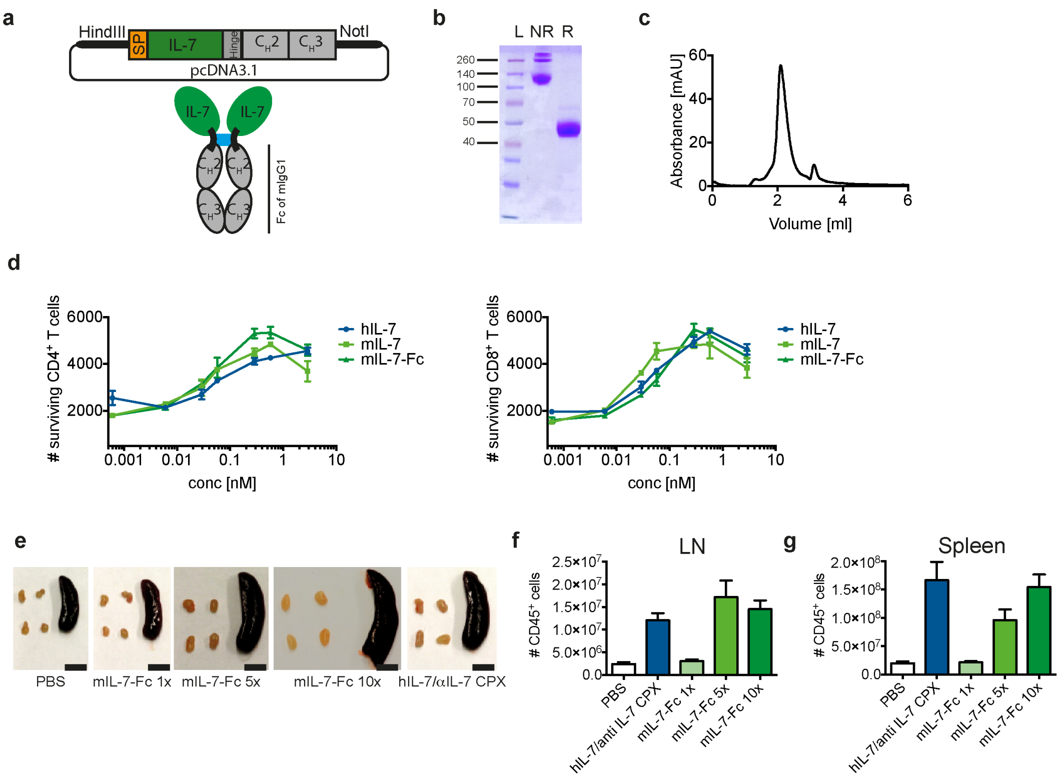

Suppl. Fig. 7. Production and characterization of mIL-7-Fc. (**a**) Schematic representation of the pcDNA3.1 coding vector (top) and the resulting dimerized protein (bottom), mIL-7-Fc, consisting of the secretion peptide (SP) and murine IL-7 linked to the hinge region and constant C_H2 and C_H3 domains of murine IgG1. (**b**) SDS-PAGE of mIL-7-Fc under non-reducing (NR) and reducing (R) conditions. Expected molecular weight for the unglycosylated protein is 41 KDa. (**c**) Size exclusion chromatography of mIL-7-Fc, showing a single peak at the expected retention volume of the dimeric fusion protein. (**d**) *In vitro* activity assay: Isolated lymphocytes were cultured with increasing concentrations of recombinant human (hIL-7), murine (mIL-7), and mIL-7-Fc (molar equivalents). After 72 hours the number of surviving T cells was quantified by FACS. The number of surviving $CD4^+$ (left) and $CD8^+$ (right) T cells is dose-dependent. 1 representative out of 4 similar experiments with 3 replicates (mean ± SEM) per treatment is shown. (**e-g**) *In vivo* activity assay: C57BL/6 mice were treated intraperitoneally (i.p.) every second day for one week with phosphate buffered saline (PBS), 1.5 μg/15 μg human IL-7/anti-IL-7 antibody complexes (hIL-7/IL-7 CPX, [1]), and 1x, 5x, and 10x molar equivalent doses of mIL-7-Fc. (**e**) Representative images of LN and spleen enlargement (scale bar = 0.5 cm). Number of $CD45^+$ cells on day 8 in (**f**) pooled brachial and inguinal LNs and (**g**) spleen. Pooled data (mean ± SEM) from all mice included in three independent experiments are shown. With the exception of the mIL-7-Fc (1x) group (n=3 mice), which was only included in the first experiment, all other groups were evaluated in 2-3 experiments (total of 7-14 mice/ per condition).

1 Boyman, O., Ramsey, C., Kim, D. M., Sprent, J. & Surh, C. D. IL-7/anti-IL-7 mAb complexes restore T cell development and induce homeostatic T Cell expansion without lymphopenia. *J Immunol* **180**, 7265-7275 (2008).

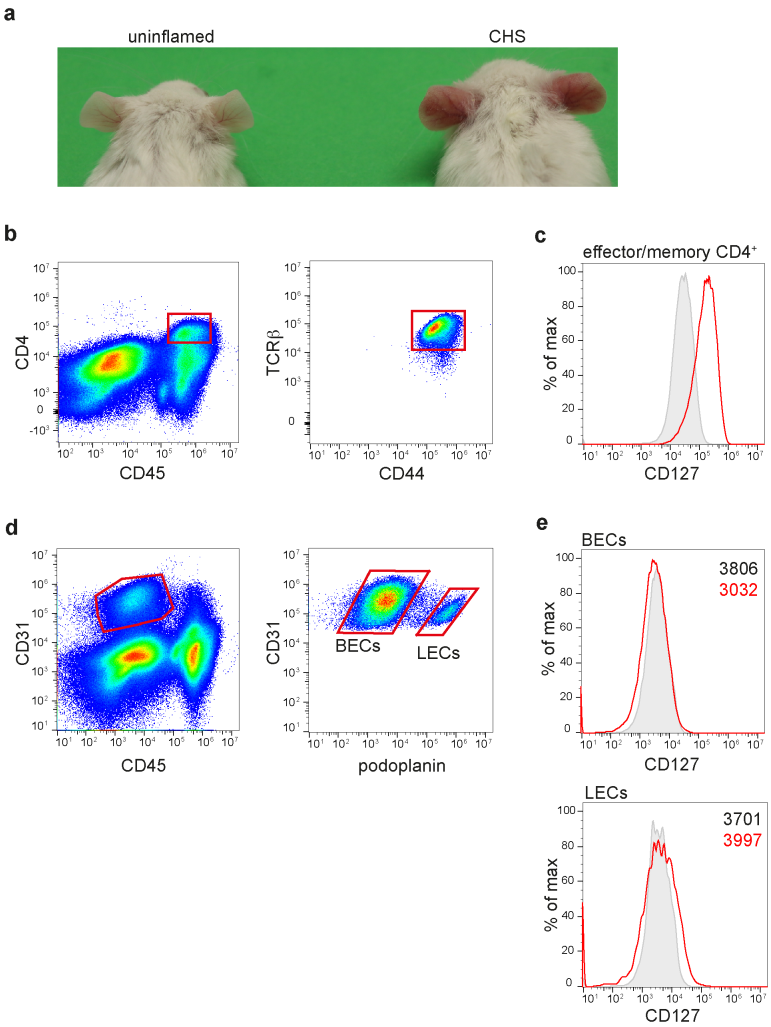

Suppl. Fig. 8. K14-VEGF-A model: Characterization of IL-7Rα (CD127) expression in CD4$^+$ T cells and endothelial cells at the onset of treatment. Hemizygous K14-VEGF-A mice were sensitized with oxazolone on the belly and paws. Five days later their ears were challenged with oxazolone to induce a CHS response. (**a**) Representative images of inflamed ears on day 7 after challenge (right, CHS), i.e. the normal onset of treatment (see Fig. 2A & Fig. 3A). On the left, an uninflamed control is shown for comparison. (**b-e**) On day 7, CD127 (IL-7Rα) expression on CD4$^+$ effector/memory T cells, BECs and LECs was determined by FACS analysis performed on ear skin single cell suspensions. (**b**) Depiction of the gating scheme used to identify CD4$^+$ effector/memory T cells (CD45$^+$CD4$^+$CD44^{hi}TCRβ$^+$) (**c**) Expression of IL-7Rα protein on CD4$^+$ effector/memory T cells. The red line indicates IL-7Rα expression, the tinted histogram shows the corresponding isotype control staining. (**d**) Depiction of the gating scheme used to identify BECs (CD45$^-$CD31$^+$podoplanin$^-$) and LECs (CD45$^-$CD31$^+$podoplanin$^+$). (**e**) Expression of IL-7Rα protein in BECs and LECs. The red lines indicate IL-7Rα expression, tinted histograms show the corresponding isotype control stainings. MFIs are written into each plot in colour (red: IL-7Rα, black: isotype control).

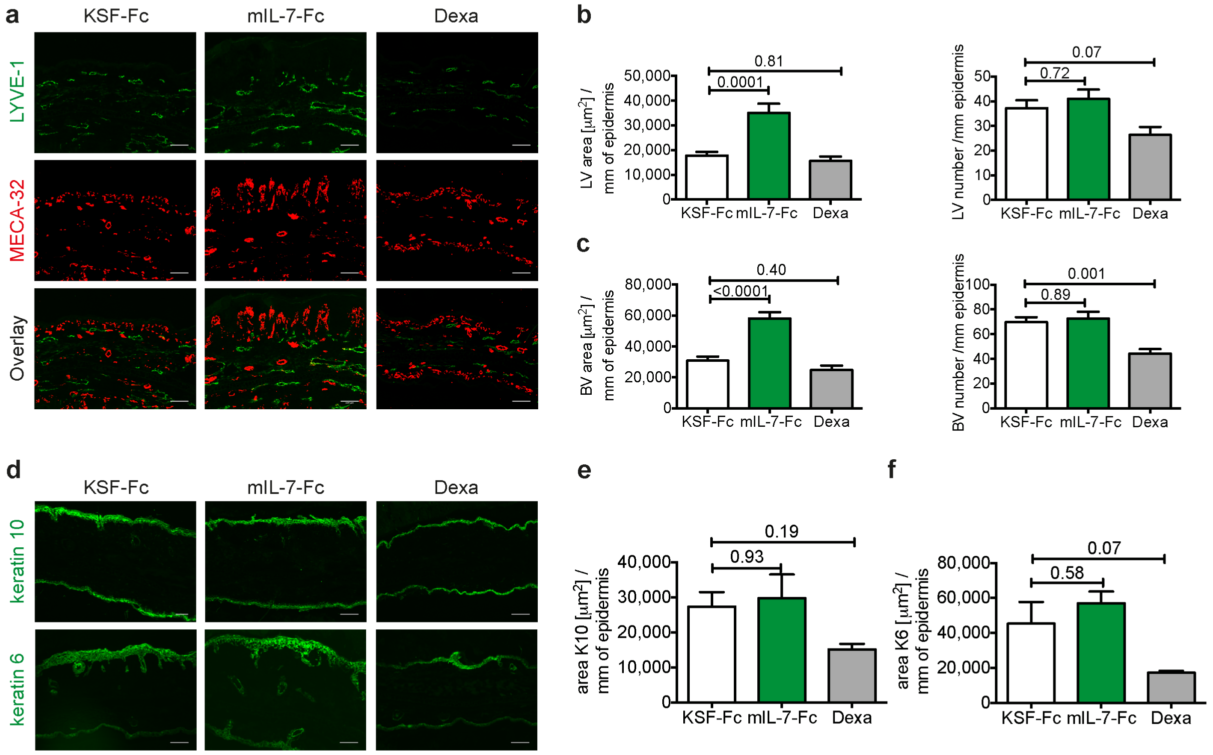

Suppl. Fig. 9. Exacerbated expansion of the vasculature but not of the epidermis in mIL-7-Fc treated mice upon oxazolone-induced inflammation. Ear sections were stained for the lymphatic marker lymphatic vessel endothelial hyaluronan receptor-1 (LYVE-1), the blood vessel (BV) marker MECA-32, and the keratin markers keratin 6 and keratin 10 on day 15 after challenge with oxazolone in K14-VEGF-A mice. Mice were treated with mIL-7-Fc, KSF-Fc, or dexamethasone as described in (Fig.2A). (**a**) Representative images of LYVE-1 and MECA-32 staining in the different treatment groups (scale bar = 100 µm). Quantification of vessel area (left) and number (right) per mm of epidermis for (**b**) LYVE-1$^+$ lymphatic vessels and (**c**) MECA-32$^+$ blood vessels. Pooled data (mean ± SEM) from 2 similar experiments with a total of 11-12 mice per group are shown. (**d**) Representative images of keratin 6 and 10 staining in the different treatment groups (scale bar = 100 µm). Keratin 6 is expressed in hyperproliferating keratinocytes and keratin 10 is a marker for keratinocyte differentiation [2]. Quantification of the area of (**e**) keratin 10 and (**f**) keratin 6 per mm of epidermis. Data (mean ± SEM) from 1 experiment with a total of 5-6 mice per group are shown.

2 Mommers, J. M., van Rossum, M. M., van Erp, P. E. & van De Kerkhof, P. C. Changes in keratin 6 and keratin 10 (co-)expression in lesional and symptomless skin of spreading psoriasis. *Dermatology* **201**, 15-20, doi:10.1159/000018422 (2000).

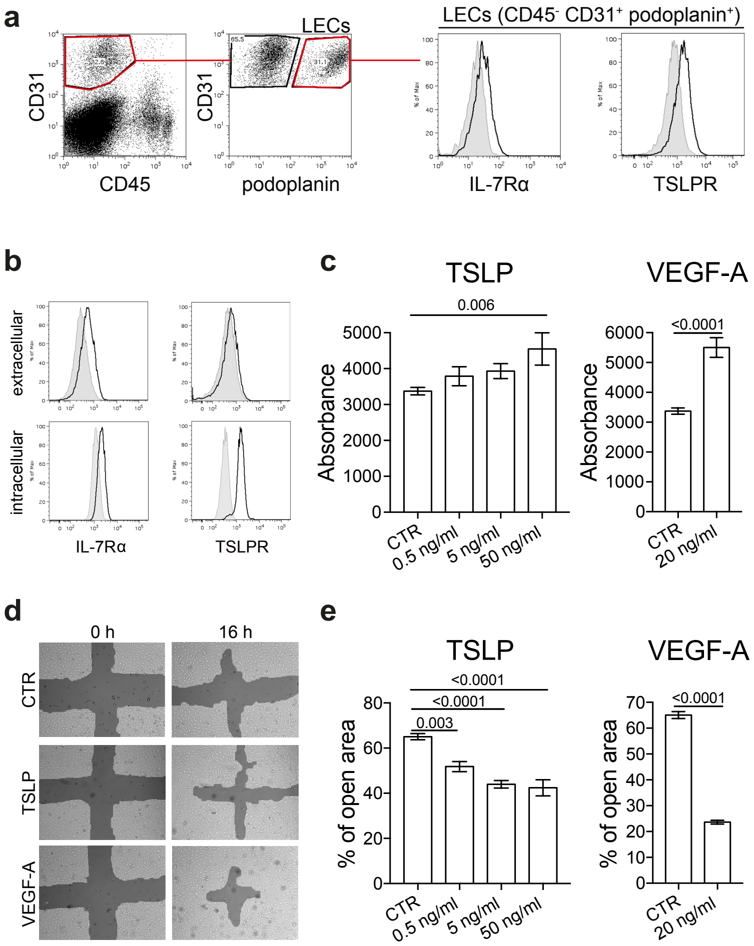

Suppl. Fig. 10. TSLPR is expressed by LECs and TSLP induces lymphangiogenic processes in cultured human LECs. (a) FACS analysis was performed on murine ear cell suspensions. (**a**) Depiction of the gating scheme used to identify LECs ($CD45^{-}CD31^{+}podoplanin^{+}$). LECs stained positive for both IL-7Rα and TSLPR protein. Representative plots from 3 different experiments are shown. (**b**). FACS analysis IL-7Rα and TSLPR expression in cultured human LECs. FACS cell surface staining (extracellular) detected low levels of IL-7Rα but virtually no TSLPR expression in cultured human LECs. Higher TSLPR expression levels were detected when performing the FACS staining on PFA-fixed and permeabilized human LECs (i.e. additionally staining for intracellular protein). Black line: candidate gene. Grey, tinted line: isotype control. Representative plots from 3 different experiments are shown. (**c**) TSLP induces LEC proliferation and migration in cultured human LECs. Cultured human LECs were treated for 3 days with TSLP or with VEGF-A. A subtle, but significant, dose-dependent increase in LEC proliferation was measured upon stimulation with TSLP. As a positive control, VEGF-A-induced proliferation was analyzed. (**d, e**) A cell-free scratch was introduced into confluent LEC monolayers and the effect of TSLP or VEGF-A on scratch closure, which represents migration of bordering LECs into the cell-free zone, was analyzed 16 hours later. (**d**) Representative images showing the effects of IL-7 and of VEGF-A on scratch closure. (**e**) Quantitative analysis of the reduction in open area induced by treatment with IL-7 or with VEGF-A. Results from 1 out of 3 similar experiments are shown in (c-e).

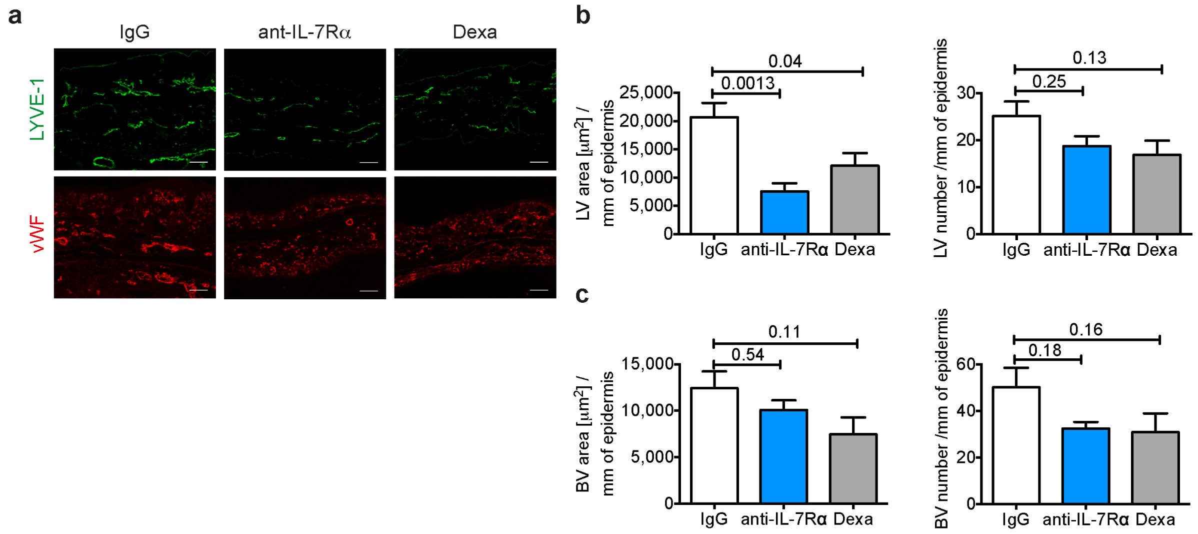

Suppl. Fig. 11. Normalization of the vasculature in anti-IL-7Rα treated mice upon oxazolone-induced inflammation in K14-VEGF-A mice. Ear sections were stained for the lymphatic marker LYVE-1 and the BV marker von Willebrand factor (vWF) on day 15 after challenge with oxazolone in K14-VEGF-A mice. Mice were treated with mIL-7-Fc, KSF-Fc, or dexamethasone as described in (Fig.3A). (**a**) Representative images of LYVE-1 and vWF staining in the different treatment groups (scale bar = 100 μm). Quantification of vessel area (left) and number (right) per mm of epidermis for (**b**) LYVE-1^{+} lymphatic vessels and (**c**) vWF^{+} blood vessels. Data (mean ± SEM) from 1 experiment with a total of 5-6 mice per group are shown.

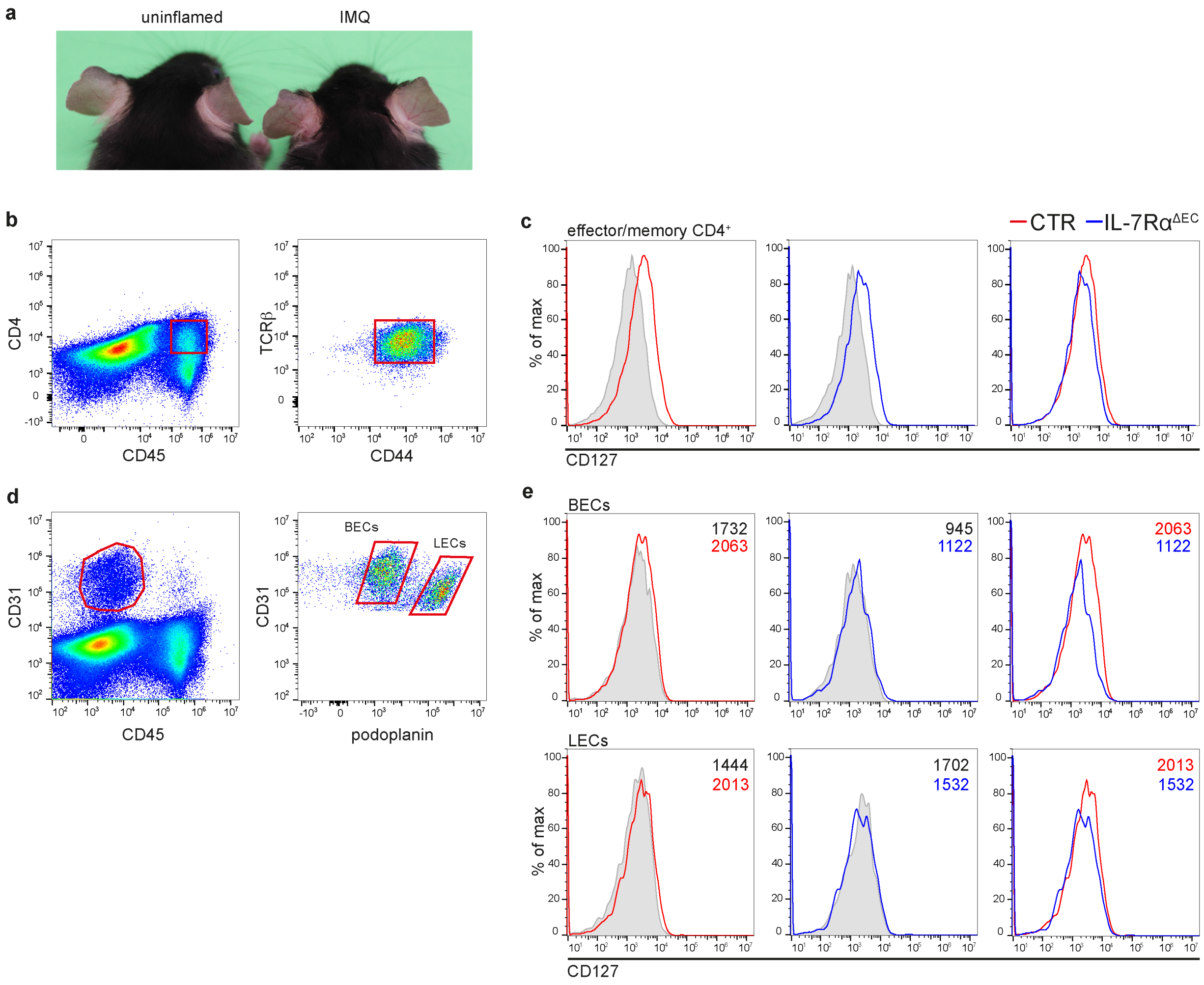

Suppl. Fig. 12. Imiquimod model: Characterization of IL-7Rα (CD127) expression in CD4^{+} T cells and endothelial cells at the onset of treatment. Imiquimod-containing cream was applied to the ears on day 1, 2, 3, 4, and 5. (**a**) Representative images of IMQ-inflamed ears of a WT mouse (right; IMQ) on day 7, i.e. the normal onset of treatment (see Fig. 4A). On the left the ears of an uninflamed WT mouse are shown for comparison. (**b-e**) FACS-based analysis of CD127 (IL-7Rα) expression on CD4^{+} effector/memory T cells, BECs and LECs in single cell suspensions generated from day 7 IMQ-inflamed ears of a IL-7Rα$^{\Delta EC}$ mouse (VE-cadherin$^{cre/cre}$ x IL-7Rα$^{fl/fl}$) and a control mouse (CTR: VE-cadherin$^{wt/wt}$ x IL-7Rα$^{fl/fl}$). (**b**) Depiction of the gating scheme used to identify CD4^{+} effector/memory T cells (CD45^{+}CD4^{+}CD44^{hi}TCRβ^{+}). (**c**) The coloured lines indicate IL-7Rα expression in IL-7Rα$^{\Delta EC}$ (blue) and control (red), tinted histograms show the corresponding isotype control stainings. (**d**) Depiction of the gating scheme used to identify BECs (CD45^{-}CD31^{+}podoplanin^{-}) and LECs (CD45^{-}CD31^{+}podoplanin^{+}). (**e**) The coloured lines indicate IL-7Rα expression in IL-7Rα$^{\Delta EC}$ (blue) and in control (CTR: red), tinted histograms show the corresponding isotype control stainings. MFIs are written into each plot in colour (red: CTR, blue: IL-7Rα$^{\Delta EC}$, black: isotype control).
